# Coccolith-calcite Sr/Ca as a proxy for transient export production related to Saharan dust deposition in the tropical North Atlantic

**DOI:** 10.1038/s41598-024-54001-3

**Published:** 2024-02-21

**Authors:** C. V. Guerreiro, P. Ziveri, C. Cavaleiro, J.-B. W. Stuut

**Affiliations:** 1 MARE - Marine and Environmental Sciences Centre/ARNET - Aquatic Research Network, Faculty of Sciences of the University of Lisbon (FCUL), Lisbon, Portugal; 2 IDL, Instituto Dom Luiz, FCUL, Lisbon, Portugal; 3 Department of Plant Biology, FCUL, Lisbon, Portugal; 4 ICREA, Catalan Institution for Research and Advanced Studies, Barcelona, Spain; 5 ICTA-UAB, Institut de Ciència i Tecnologia Ambientals—Universitat Autònoma de Barcelona, Barcelona, Spain; 6 Marine Geology and Georesources (DivGM), IPMA – Portuguese Institute for Sea and Atmosphere, Lisbon, Portugal; 7 Department of Ocean Systems, NIOZ Royal – Netherlands Institute for Sea Research, Den Burg, The Netherlands; 8 Faculty of Earth and Life Sciences, Vrije Universiteit (VU), Amsterdam, The Netherlands

**Keywords:** Biogeochemistry, Climate sciences, Ecology, Limnology, Ocean sciences

## Abstract

Atmospheric dust deposition can modulate the earth’s climate and atmospheric CO_2_ through fertilising the ocean (nutrient source) and by accelerating the biological carbon pump through fuelling the ballasting process. To distinguish the biogeochemical effects of Saharan dust with respect to fertilization and ballasting, and to gain a broader perspective on the coccolith calcite Sr/Ca in relation to the drivers of coccolith export production, we determined the coccolith-Sr/Ca from a one-year (2012–2013) time-series sediment trap record in the western tropical North Atlantic (M4—49°N/12°W). High Sr/Ca were linked to enhanced export production in the upper part of the photic zone, most notably under windier, dry, and dustier conditions during spring. Attenuated Sr/Ca in the autumn probably reflect a combination of lower Sr-incorporation by dominant but small-size placolith-bearing species and the presence of “aged” coccoliths rapidly scavenged during a highly productive and usually fast export event, likely added by (wet) dust ballasting. Higher Sr/Ca observed in the large coccolith size fractions support the existing notion that larger-sized coccolithophores incorporate more Sr during calcification under the same environmental conditions. The presence of the abnormally Sr-rich species *Scyphosphaera apsteinii* is also shown in the separated large fraction of our Sr/Ca seasonal data.

## Introduction

Increasing dust outbreaks triggered by climate-induced land desertification^1,2^ have the potential of counteracting the negative effects from ongoing ocean stratification through providing an alternative source of nutrients (such as phosphorus and iron) fertilising primary production in an ever-warmer ocean^3–6^. At the same time, dust is also a source of mineral ballast which is critical for enhancing the export and sequestration of particulate organic carbon (POC) by increasing the sinking velocities of carbon-enriched material produced by phytoplankton and zooplankton in the upper ocean^7^.

Recent studies report on the influence of Saharan dust deposition as sporadically stimulating the productivity and export fluxes of coccolithophores (Haptophyta) (consult the Glossary in the Supplementary Material) in the heavily stratified tropical North Atlantic^8–10^. Coccolithophores are a biogeochemically important group of phytoplankton that can intracellularly biomineralize CaCO_3_ scales (coccoliths) which are secreted onto the cell surface to collectively form a calcitic exoskeleton (coccosphere)^11^. This ability contributes to some of the highest export fluxes of particulate inorganic carbon (PIC) from the photic zone to the deep sea^12–15^. Being primary producers, coccolithophores act as a CO_2_ sink, since they fix CO_2_ during photosynthesis and provide calcitic ballasting minerals which facilitate the export and deep-sea sequestration of POC^13,16,17^, similar to dust particles. In parallel, as calcification incorporates carbon into calcite, it reduces the availability of the carbonate ion in the surface ocean while releasing CO_2_, thereby causing coccolithophores to also act as CO_2_ source^13^.

In a series of sediment-trap studies across the tropical North Atlantic downwind of NW Africa, Guerreiro et al.^8,9^ reported on the occurrence of pulsed export production events by the opportunistic coccolithophores *Emiliania huxleyi* and *Gephyrocapsa oceanica* in the western part of the ocean basin (mooring site M4 at 12°N/49°W), which were linked to Saharan dust deposition. Both events were marked by a striking increase of POC and of bSiO_2_/CaCO_3_ ratios (i.e., proxy for diatom *versus* biogenic CaCO_3_ export production), and by a decrease of the coccolith-PIC/POC molar ratios (i.e., proxy for coccolithophore contribution to the PIC/POC ratio), suggesting that dust-born nutrient input had contributed to stimulate the biological carbon pump^17,18^. More recently, Guerreiro et al.^10^ reported on enhanced abundance (cells/L) of *E. huxleyi* and *G. oceanica*, and of N_2_-fixing filamentous cyanobacteria *Trichodesmium* sp. in the photic zone of the tropical NE Atlantic (~ 28°W), in response to Saharan-driven input of iron and phosphorous. While this recent finding supports the dust-related coccolith export productivity events above, it remains an open question whether they mostly reflected a nutrient-stimulated ecological (growth) response or if they were the result of increased export efficiency related to dust- and coccolith-ballasting (see^17,19^).

**Figure 1:**
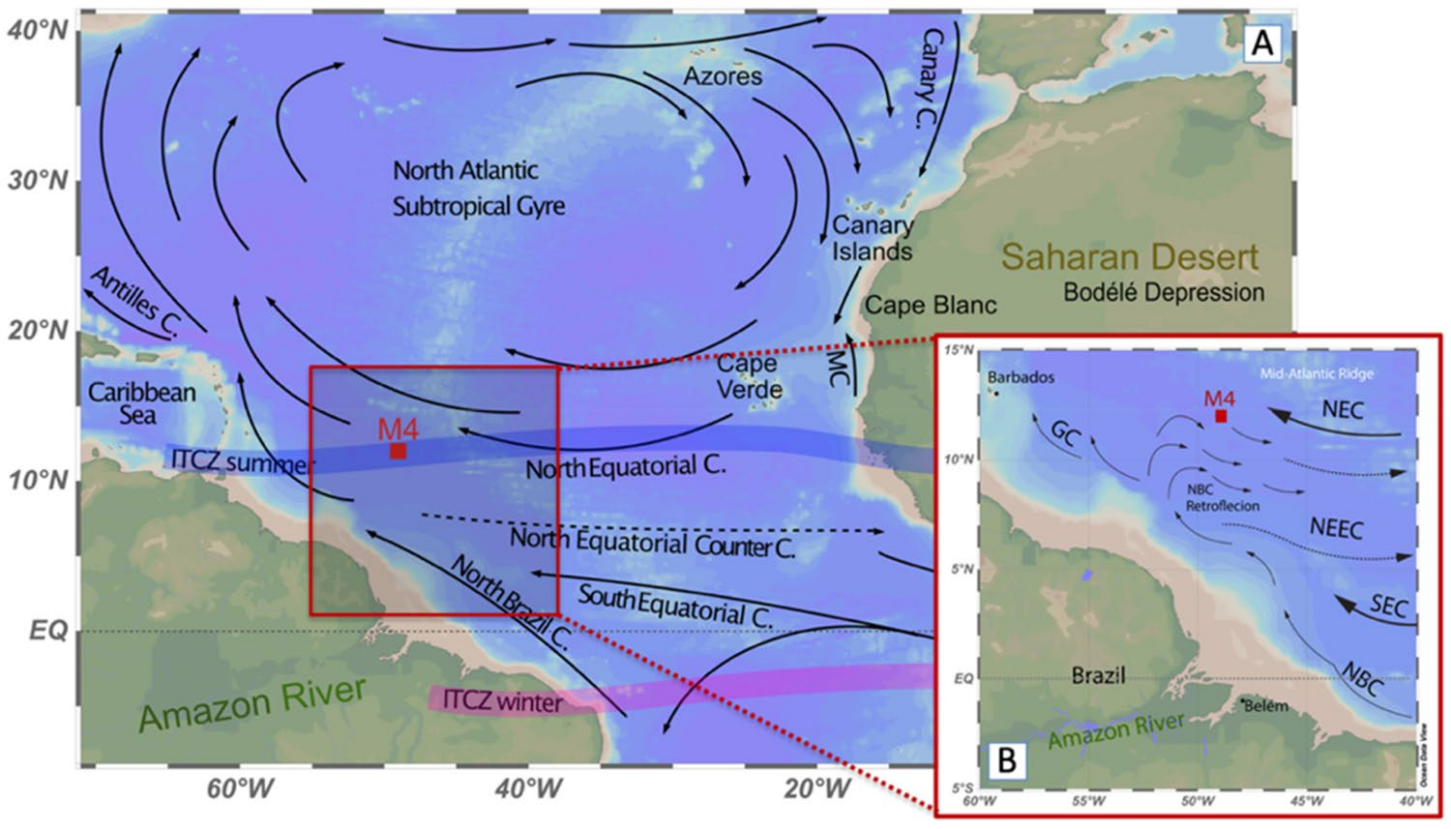
Location of trap mooring site M4 (**A**), and a schematic representation of the main surface currents in the equatorial Atlantic Ocean, with the inset showing the seasonal eastward retroflection of the North Brazilian Current (NBC) during boreal summer (**B**) (adapted from^8,36^).

To answer this question, we have quantified the coccolith-Sr/Ca ratio from the same samples collected at trap site M4 (Fig. 1). Our approach is based on previous studies reporting higher Sr partitioning (i.e., higher amount of Sr incorporated into the calcite) as directly proportional to the coccolith calcification rate which, in turn, is a function of the coccolithophores’ growth rate (e.g.^20–23^). Because Sr/Ca ratios of seawater vary by less than 2% globally, and since the link between coccoliths Sr partitioning and their calcification rates is consistent with the control of Sr partitioning by calcification rate in abiogenic calcites^22,23^, substantial variations in Sr/Ca (> 20%) are assumed to reflect growth-related Sr partitioning in coccolith calcite. Basically, the faster coccolithophores grow, the faster they calcify, resulting in more Sr being incorporated into the calcite lattice of their coccoliths^20,24^. In the same way, nutrient limitation in coccolithophores has been reported to reduce the uptake of Sr compared to Ca into the calcifying vesicle, thereby decreasing the coccolith Sr/Ca ratios^25–28^. Therefore, variations in coccolith-Sr/Ca ratios can be used as proxies for nutrient-stimulated growth rates of coccolithophores^21,29–31^. Previous sediment-trap records in the Sargasso Sea and Arabian Sea^30^, and in the Eastern Mediterranean^32^ have already provided relevant indications about nutrient limitation of coccolithophore in situ productivity versus coccolith export efficiency *out* of the photic zone based on coccolith Sr/Ca ratios. Auliaherliaty et al.^33^ further demonstrate that Sr/Ca are not impacted by bacterial influences on algal physiology, thereby reinforcing the robustness of this ratio as a productivity proxy.

Our study is based on new data of coccolith Sr/Ca signatures measured in sinking coccolith assemblages collected at sediment trap site M4 from October 2012 to October/November 2013, in relation to published data from the same trap/period, i.e., species-specific fluxes of coccoliths and coccolith-CaCO_3_^8,17^; fluxes of carbonate, organic matter, biogenic silica—bSiO_2,_ and mineral dust^34,35^. The main goal is to gain a broader perspective on the coccolith-Sr/Ca in relation to the drivers of coccolith export production, by linking the surface production with the export particle fluxes towards distinguishing the biogeochemical effects of Saharan dust acting as both a fertilizer and/or as ballast.

## Material and methodology

### Sediment trap sampling

Our study is based on time-series particle flux material collected by sediment-trap mooring M4 (12°N/49°W) at intervals of 16 days during one year (from 4 October 2012 to 7 November 2013) at 1200 m water depth in the western tropical North Atlantic (Fig. 1). Details of the mooring equipment, the deployment/recovery of the sediment trap, and the treatment of the recovered sample bottles are described in the cruise report^37^. Sediment-trap samples were initially wet-sieved over a 1 mm mesh, wet-split into five aliquot subsamples using a rotary splitter (WSD-10; McLane Laboratories), washed to remove the HgCl_2_ and salts, and centrifuged. Average weight differences between replicate aliquots were within 2.4% (SD = 2.2), with 87% of all samples differing < 5% between splits (detailed procedure in^34^).

Previous studies have already extensively described the oceanographic and meteorological settings, as well as the seasonal patterns of particle fluxes at mooring trap site M4 during the sampling period studied here^8,34,35^. A summary of environmental background at the location of mooring M4 is provided in the Supplementary Material.

### Coccolith- and coccolith-CaCO_3_ flux analysis

Data of species-specific coccolith- and coccolith-CaCO_3_ fluxes presented in this study are from^8,17^. These studies are based on a minimum of 500 coccoliths counted from an arbitrarily chosen transect and each coccolith was identified to the lowest taxonomic level possible at 3000 × magnification using a Zeiss DSM 940A SEM at 10 kV of accelerating voltage. This number of counts per sample is well above the recommended minimum of 300 specimens for studies focused on quantifying species with > 3% of the assemblage, corresponding to a 95% confidence level^38^. The UPZtaxa/LPZtaxa ratio presented in Fig. 4c, used as a proxy for inferring variations in the depth of the nutricline, is calculated by dividing the fluxes produced by typically upper photic zone (UPZ) species *Emiliania huxleyi* and *Gephyrocapsa* spp., by the fluxes produced by lower photic zone (LPZ) species *Florisphaera profunda* and *Gladiolithius flabellatus*^8,9^.

The coccolith-derived CaCO_3_ export fluxes were determined using the mass equation of^39^, according to which the coccolith mass of distinct species is expressed as: **Coccolith calcite (pg) = 2.7 × Ks × l**^**3**^, in which 2.7 = density of calcite (CaCO_3_); ks = shape constant; l = coccolith size (mostly distal shield length). The size of around 3500 coccoliths were measured from most species present in the samples from trap M4 using the same SEM. A detailed description of the quantification of the coccolith-calcite masses and coccolith-CaCO_3_ fluxes is presented in^17^.

### Quantification of the coccolith-Sr/Ca ratios

The coccolith Sr/Ca sample processing and analyses were adapted from^29^ and^31^, focusing on measuring Sr/Ca ratios from different enriched coccolith size fractions which had been previously separated via repeated decanting prior to the chemical analysis. This approach aims to obtain different size fractions dominated by distinct coccolith species with distinct calcite mass and sinking rate. Different species can indeed have different coccolith calcite partitioning of Sr and amplitudes of Sr/Ca variation (e.g.,^40–42^). The laboratory procedure is further described in the Supplementary Material. We present the Sr/Ca ratios determined from the bulk fraction (< 20 µm) and from the three coccolith size-fractions (> 6 µm, 3–6 µm and < 3 µm), which we compared to published data concerning the total and species-specific coccolith- and coccolith-CaCO_3_ export production from the same samples. In addition to the species-specific CaCO_3_ contribution to the < 20 µm bulk fraction, we also show their CaCO_3_ contribution for a series of coccolith size fractions obtained from trap samples U2, U7, U12, U14, U18, U21 and U24, which were selected for microscopic inspection. The total number of counts performed in the coccolith size fractions (150 < n < 390 liths) was high enough to ensure the quantification of rare species within the assemblage within a 95% confidence level^38^ which often contribute disproportionally high amounts of carbonate to the assemblage despite of its low abundance. Such was particularly the case for the very large *Scyphopshaera apsteinii* for which we determined the following 95% confidence intervals of coccolith counts for the fractions where this species was found: [0–3] for the intermediate fraction of U2, and for the large fractions of U12, U18 and U21; and [0–6] for the large fraction of U14. Despite reported evidence on ocean temperature affecting the partitioning of Sr into the coccolith calcite (~ 0.03 mmol/mol increase per °C increase^24,43^, the temperature changes during our study (up to 3 °C) can be neglected compared to the observed Sr/Ca changes (i.e., a change of 3 °C entails a maximum change of 0.9 mmol/mol in the range (0.7–12.6 mmol/mol), average (4.1 mmol/mol) and standard deviation (0.7) of our Sr/Ca results. Table 1 shows the Sr/Ca results obtained from this study. For the calculation of the normalized Sr/Ca ratios presented in Fig. 4a, we first determined a “Sr/Ca anomaly” by subtracting the Sr/Ca annual mean to the Sr/Ca ratio of each sample; and then we divided each Sr/Ca anomaly by the Sr/Ca annual mean deviation to obtain the normalized values.

**Table 1:** Coccolith Sr/Ca (mmol/mol) results from the chemical elemental analysis.

Trap M4	Start date	End date	Mid date	Coccolith Sr/Ca (m mol/mol)			
				< 20 μm	< 3 μm	3–6 μm	> 6 μm
U1	19/10/2012	04/11/2012	27/10/12	2.7	–	–	6.58
U2	04/11/2012	20/11/2012	12/11/12	1.6	1.22	1.00	2.94
U3	20/11/2012	06/12/2012	28/11/12	1.2	1.40	0.72	1.62
U4	06/12/2012	22/12/2012	14/12/12	1.4	1.03	0.79	1.94
U5	22/12/2012	07/01/2013	30/12/12	2.7	1.56	2.04	7.78
U6	07/01/2013	23/01/2013	15/01/13	2.7	1.56	1.91	8.55
U7	23/01/2013	08/02/2013	31/01/13	3.3	1.72	2.52	9.06
U9	24/02/2013	12/03/2013	04/03/13	3.0	–	–	–
U10	12/03/2013	28/03/2013	20/03/13	2.2	2.06	–	1.85
U11	28/03/2013	13/04/2013	05/04/13	2.6	1.48	2.29	8.80
U12	13/04/2013	29/04/2013	21/04/13	4.1	2.46	3.86	7.03
U13	29/04/2013	15/05/2013	07/05/13	4.6	1.57	2.91	9.90
U14	15/05/2013	31/05/2013	23/05/13	5.4	2.01	5.66	11.73
U15	31/05/2013	16/06/2013	08/06/13	3.9	1.78	3.87	8.98
U16	16/06/2013	02/07/2013	24/06/13	4.0	1.78	2.83	9.51
U17	02/07/2013	18/07/2013	10/07/13	5.0	1.61	3.18	12.56
U18	18/07/2013	03/08/2013	26/07/13	4.9	1.84	3.72	11.91
U19	03/08/2013	19/08/2013	11/08/13	4.0	1.94	3.11	12.09
U20	19/08/2013	04/09/2013	27/08/13	4.6	1.70	4.90	10.69
U21	04/09/2013	20/09/2013	12/09/13	4.3	1.57	3.92	10.73
U22	20/09/2013	06/10/2013	28/09/13	3.5	1.81	2.99	8.63
U23	06/10/2013	22/10/2013	14/10/13	3.8	1.65	–	10.10
U24	22/10/2013	07/11/2013	30/10/13	2.2	1.76	1.37	4.55

### Satellite remote sensing

Time series of meteorological and hydrological data obtained from satellite remote sensing were used to provide a complementary perspective on the environmental conditions during the in-situ sampling period (datasets details provided in Table I—Supplementary Material). The mixed-layer depth (MLD, defined as being the deepest ocean layer affected by wind-forced turbulent mixing) was used as an indicator of seasonal environmental variations related to the Intertropical Convergence Zone (ITCZ) in terms of ocean thermal stratification vs. wind-forced water cooling and mixing (i.e., < MLD indicates weaker trade winds due to a greater influence of the ITCZ on trap site M4^9^. Sea surface salinity (SSS) was used as an indicator of salinity variations linked to the seasonal entrainment of Amazon River water during the sampling period^8^. Satellite data were retrieved using a 2° × 2° latitude–longitude area around the location of trap M4 and averaged for each sediment trapping interval. The 2° × 2° box, corresponding to ~ 108 × 108 N mi (1° =  ~ 59 N mi), was taken as representative of the catchment area of a sediment trap deployed at 1200 m depth, given the sinking speed for marine phytoplankton and algal aggregates (e.g.,^8,44^).

### Statistical analysis

In order to investigate the relationship between the Sr/Ca ratios and the environmental conditions during the sediment trap period, a Principal Component Analysis (PCA, correlation mode, by PAST-4.11) was performed upon a data matrix with species-specific CaCO_3_ fluxes, Sr/Ca ratios of all studied coccolith size fractions, fluxes of organic matter, biogenic silica (bSiO_2_) and mineral dust, and with remotely sensed Chlorophyll-a (Chla), SSS and MLD as columns (n = 18 variables). Only the samples with Sr/Ca data available for all size fractions were considered (n = 20 cases). A Spearman correlation coefficient matrix was also built upon the same data matrix for assessing the statistical significance of the correlations obtained from the PCA, using a default p-level of 0.05. All the results from the statistical analysis are presented in the Supplementary Material.

## Results

### Coccolith CaCO_3_ size fraction separation for Sr/Ca analyses

**Figure 2:**
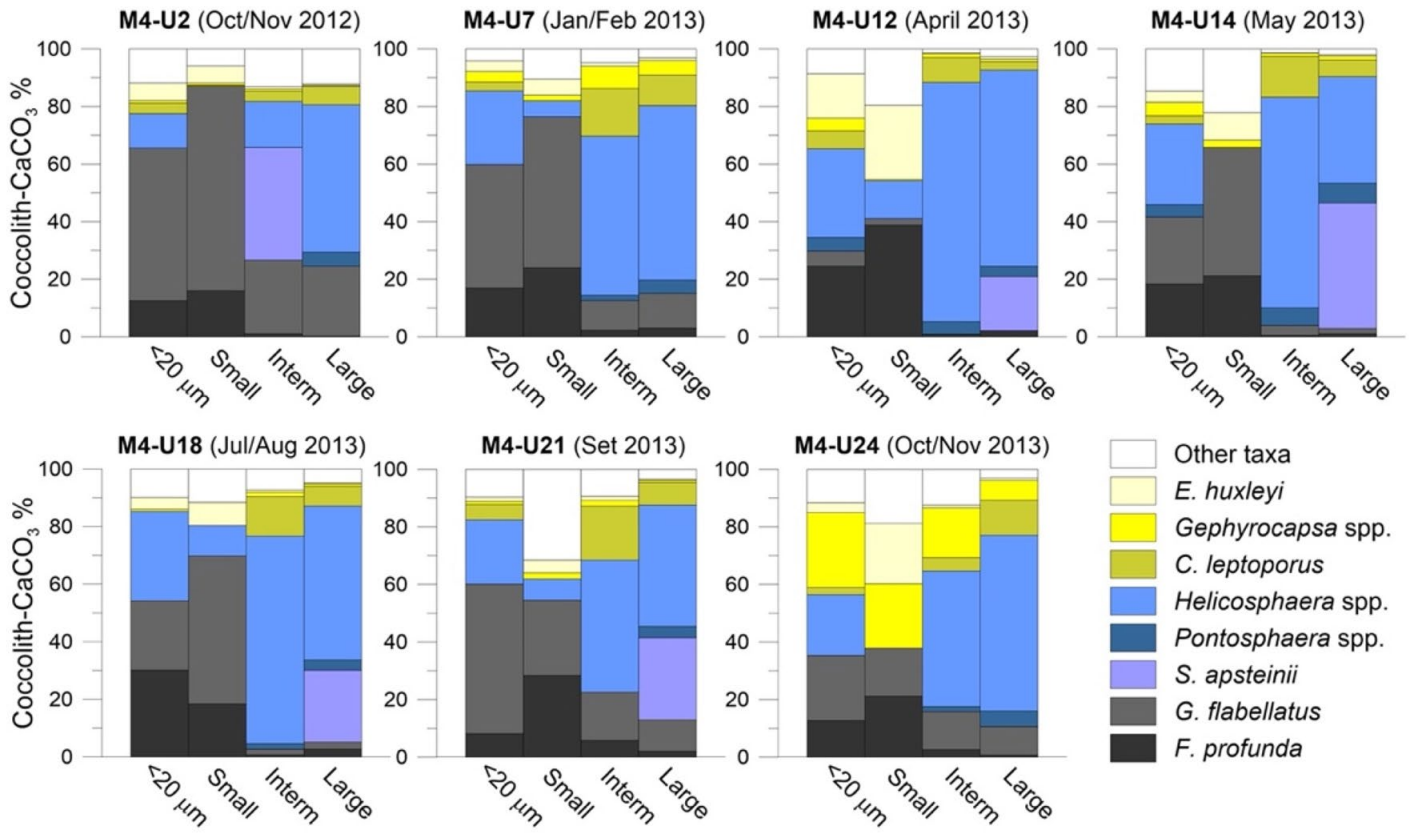
Species-specific coccolith-CaCO_3_ contribution (%) in the bulk fraction (< 20 μm) and coccolith size-fractions (small < 3 µm; intermediate 3–6 µm; and large > 6 µm), from selected sediment trap M4 samples U2, U7, U12, U14, U18, U21 and U24.

The Sr/Ca was obtained from three coccolith size fractions, each characterized by CaCO_3_ content driven by a few species of similar coccolith size (Fig. 2). The bulk fraction (< 20 µm) overall mimicked the seasonal variation of the original coccolith sinking assemblages and related coccolith-CaCO_3_ reported by^8,17^. The small fraction (< 3 µm) was dominated (38–87%) by CaCO_3_ from deep-dwelling species *Gladiolithus flabellatus* and *F. profunda.* The intermediate (~ 3–6 µm) and large fractions (> 6 µm) were dominated by carbonate produced by *Helicosphaera* spp. (up to 83%) followed by *Scyphosphaera apsteinii* (up to 44%), *Calcidiscus leptoporus* (up to 19%) and *Pontosphaera* spp. (up to 7%). This high carbonate contribution by *S. apsteinii* to the large size fraction results from its unusual large size found in M4 samples, thereby contributing a disproportionally high percentage compared to its low relative abundance (Fig. I in the Supplementary Material). Note that samples U12, U14, U21 and U24 show that *E. huxleyi*, *Gephyrocapsa* spp. and the “other taxa” significantly increased their carbonate contributions in the small fraction (up to 26%, 22% and 32%, respectively), while *Gephyrocapsa* spp. also contributed to the intermediate/large fractions of samples U7 (8% and 7%, respectively) and U24 (17% and 7%, respectively) (Fig. 2).

### Seasonal distribution of the Sr/Ca ratios

**Figure 3:**
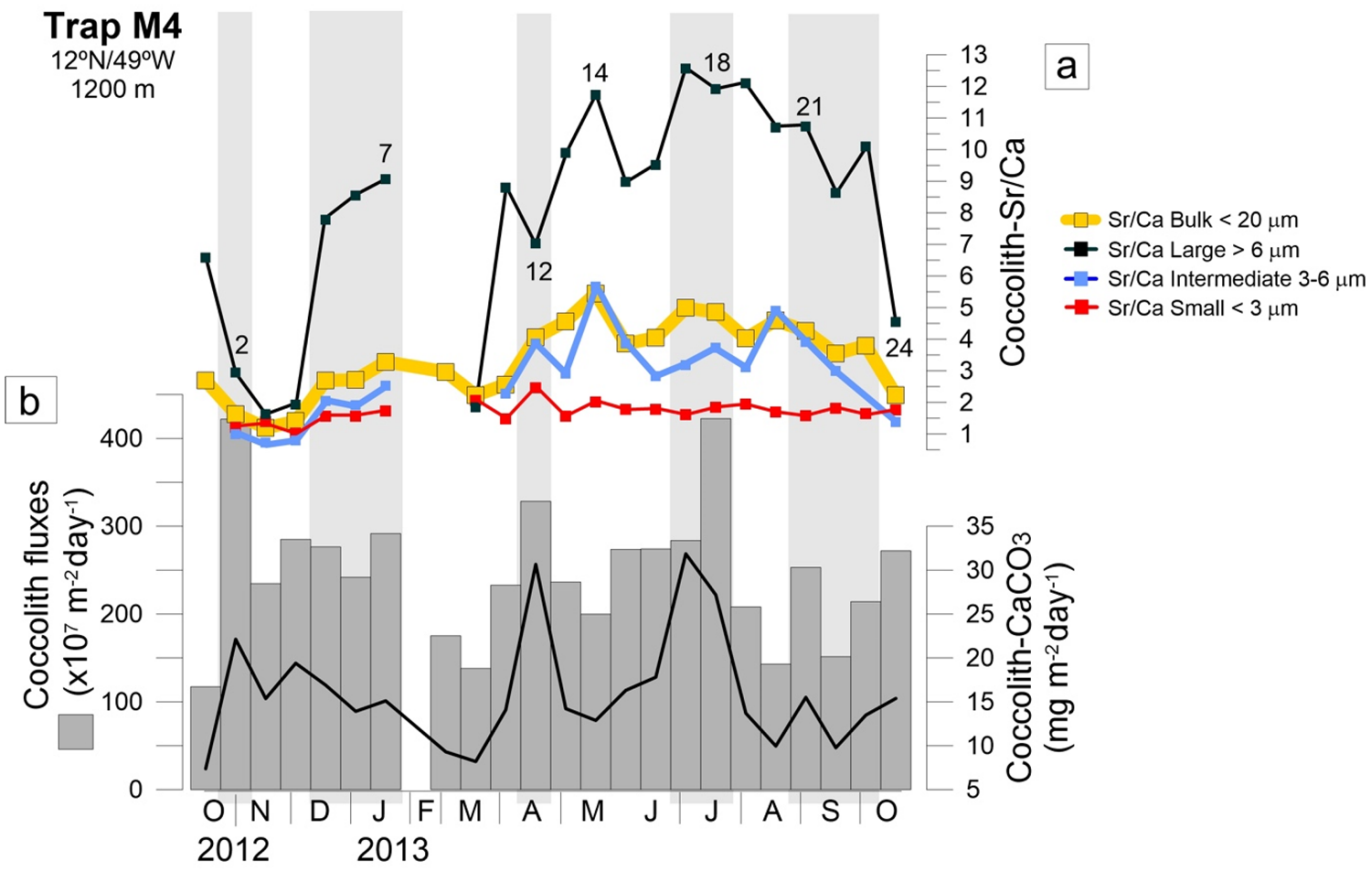
(**a**) Seasonal variation of the coccolith-Sr/Ca ratios in the bulk fraction (< 20 μm) and in the coccolith size fractions (small < 3 µm; intermediate 3–6 µm; large > 6 µm); (**b**) total coccolith- and coccolith-CaCO_3_ fluxes^8,17^ from sediment trap M4. Numbers refer to samples U2, U7, U12, U14, U18, U21 and U24, for which we performed a taxonomic analysis of all the studied coccolith size fractions (shown in Fig. 2). The light grey vertical bars indicate the periods during which co-increase in biogenic particle fluxes and Sr/Ca ratios was observed.

Here we report on the variation of the Sr/Ca for the different size fractions along the entire sediment trap period, i.e., from boreal autumn 2012 (October) until boreal autumn 2013 (October–November). We found generally higher ranges of Sr/Ca in the large fraction (> 6 µm; 1.6–12.6), followed by the bulk (< 20 µm; 1.2–5.4) and intermediate-size fractions (3–6 µm; 0.7–5.7), and finally the small size fraction (< 3 µm) with the lowest range (1–2.5). The bulk fraction revealed enough sediment to measure Sr/Ca ratios for all the analysed samples, while the small, the intermediate and the large size fractions occasionally did not (i.e., 2/23 of the samples for the small fraction; 4/23 of the samples for the intermediate-size fraction, and 1/23 of the samples for the large-sized fractions (Table 1 and Fig. 3).

**Figure 4:**
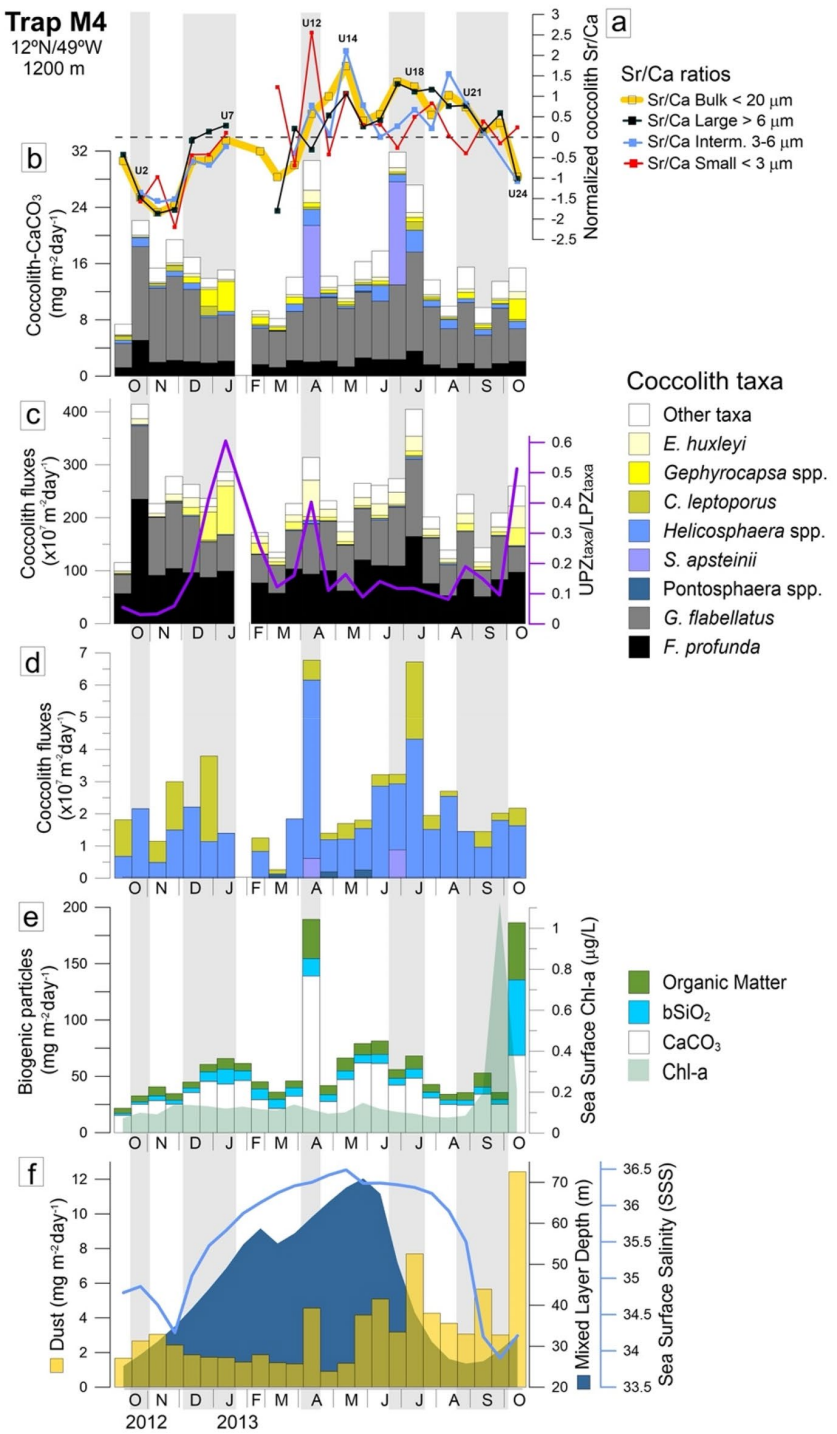
(**a**) Normalized coccolith-Sr/Ca ratios from the bulk fraction < 20 µm (light orange line) and coccolith size fractions (small, intermediate and large size fractions—red, blue and black lines, respectively); (**b**) Coccolith-CaCO_3_ fluxes produced by the main coccolithophore taxa found in the coccolith size fractions (flux data from^17^; (**c**) coccolith fluxes of the previous taxa and UPZtaxa/LPZtaxa ratio (purple line) used as a proxy for nutricline depth dynamics^8,9^; (**d**) detail of coccolith fluxes by large sized but less productive taxa (*Helicosphaera* spp. *C. leptoporus*, *S. apsteinii* and *Pontosphaera* spp.); (**e**) sea surface Chl-a concentrations, fluxes of biogenic particles (CaCO_3_, bSiO_2_ and organic matter—data from^34^; and (**f**) fluxes of mineral dust (orange—data from van der Does et al.^35^), mixed layer depth (blue) and sea surface salinity obtained from satellite remote sensing (data from^8^). Numbers refer to samples U2, U7, U12, U14, U18, U21 and U24, in which we performed a taxonomic analysis of the bulk fraction (< 20 μm) and of the coccolith small, intermediate, and large size fraction (shown in Fig. 2). The light grey vertical bars indicate the periods during which co-increase in particle fluxes and Sr/Ca ratios was observed.

Normalized Sr/Ca ratios revealed an overall similar seasonal pattern, generally higher and above the annual mean during spring and summer for all size fractions, despite some differences in their individual seasonal variation (Fig. 4a). All fractions started with Sr/Ca below the annual average in October 2012 (U1) from which it dropped significantly to a minimum in late November 2012 (bulk, intermediate and large fractions of sample U3) and in early December (small fraction of U4), further increasing to values within the annual average in January 2013 (U7). While there is no Sr/Ca data available for February and early March, the Sr/Ca of the bulk and large fractions was much lower in late March (U10) compared to January, dropping again to values below the annual mean. By contrast, the small fraction recorded a high Sr/Ca ratio during this period. Between April and early October 2013, all fractions recorded higher albeit variable Sr/Ca ratios, usually above the annual mean. Most notable increases occurred in mid-April (small fraction of U12), followed by late-May (bulk and intermediate fractions of U14), July (bulk and large fractions of U17-U18) and late August (bulk and intermediate fractions of U21). Towards the end of the sediment trap time-series, all fractions decreased to Sr/Ca ratios below the annual mean, except the small fraction which slightly increased in October–November (U24) (Fig. 4a).

Of the three studied coccolith size-fractions, the small-size fraction showed a slightly more variable seasonal pattern, particularly during the winter-spring transition. This was also the only fraction registering an increase during the dust-related productivity event in October–November 2013 (sample U24, Fig. 3).

## Discussion

### Sr/Ca record: export production driven by dust fertilization vs dust-ballasting

Our observations suggest that the Sr/Ca ratio was more efficient at tracking coccolithophore enhanced productivity occurring in the upper photic zone at site M4, as shown by its co-increase during three events of pulsed flux maxima by more opportunistic placolith-bearing species in the heavily stratified western tropical North Atlantic^17^. Such events, reported as superimposing to a yearlong predominantly tropical assemblage dominated by deep-dwelling species *F. profunda* and *G. flabellatus* (Fig. 4c), were evidenced by the striking increase of the UPZtaxa/LPZtaxa ratio in winter (January–U7), spring (April–U12) and autumn of 2013 (October–November–U24; Fig. 4c,e). Links between Sr/Ca ratios and these high productivity events are also shown in results from the PCA and Spearman correlation analysis in Tables II and III, and Fig. II of the Supplementary Material.

Increased normalized Sr/Ca ratios in all coccolith size fractions during the winter event confirm what has been hypothesized as a biogeochemical response to some degree of nutrient-enrichment driven by seasonal cooling and deepening of the MLD, typical of winter (Fig. 4a,f). While *Gephyrocapsa* spp. were the main players in this event, as also evidenced by their slightly higher carbonate contributions in all coccolith size fractions from sample U7 (Fig. 2)^34^ also report a modest but noticeable increase in the fluxes of all biogenic particles, particularly of carbonate (Fig. 4b–e).

As the cool, dry, and windy conditions of January continued to persist, and even intensify, towards spring, the striking Sr/Ca increase in the small fraction of sample U12 clearly reflects a biogeochemical response of the UPZ coccolithophore community to short-term nutrient-enrichment, as illustrated by the increased total coccolith- and coccolith-CaCO_3_ fluxes (Figs. 3 and 4b,c). Deeper MLDs during this period suggest an even greater wind-forced mixing of the upper ocean compared to January, to which nutrient supply by Saharan dust also contributed, as suggested by the increase of mineral dust fluxes (Fig. 4e,f;^35^). While *E. huxleyi* was the most productive species, other taxa increased as well, including large-sized species *S. apsteinii* and *Helicosphaera* spp., and more intermediate-sized species within the “other taxa” group, in line with the observed co-increase of the normalized Sr/Ca ratios of the bulk and intermediate-size fractions (Fig. 4a,c). The striking flux increase of all biogenic particles, including organic matter (Fig. 4e), as well as planktic foraminifera (data not shown) which are unlikely to be affected by export efficiency and have a smaller statistical funnel (see^31^), provide additional evidence that the Sr/Ca peaks in spring reflect a real dust-related production event at the location of trap M4.

These observations agree with previous studies reporting maximum Sr/Ca under similar or even cooler ocean conditions; e.g., Stoll and Schrag^20^ report on high Sr/Ca ratios coinciding with highest growth rates at temperature minima in the equatorial Pacific; Stoll and Ziveri^30^ report on Sr/Ca variations generally matching seasonal trends in coccolith fluxes of several species from sediment traps in Bermuda and the Arabian Sea, which were also consistent with inferred seasonal variations in coccolithophore surface productivity during upwelling- and monsoon-related nutrient entrainment.

By contrast, and despite the multi-proxy evidence of enhanced export productivity in autumn, the Sr/Ca of most of the studied fractions obtained from sample U24 significantly dropped (Figs. 3a and 4a), with the small fraction being the only exception as evidenced by its ca. 42% increase considering the small-fraction Sr/Ca range of 1–2.5 for the studied year; Table 1). Guerreiro et al.^8^ report this event as reflecting a response of opportunistic taxa including *E. huxleyi*, *G. oceanica* and diatoms to surface nutrient enrichment derived from seasonal entrainment of Amazon water combined with wet deposition of Saharan dust (consult the Glossary in the Supplementary Material). This is clearly evidenced by the pulsed flux maxima of coccoliths and coccolith-CaCO_3_ of *E. huxleyi* and *G. oceanica*, and of organic material and bSiO_2_, coinciding with a striking SSS drop to values < 34.5 and with the maximum dust flux at site M4 (Fig. 4b,c,e,f).

To explain why dust-related pulsed productivity was linked to enhanced Sr/Ca ratios in spring but less so in autumn, we propose a few mechanisms. First, it could be reflecting a change in species composition between the two periods, with much lower Sr being incorporated by small-sized *E. huxleyi* and *G. oceanica*, which were the main coccolith-CaCO_3_ contributors to all Sr/Ca size fractions during the autumn event (Figs. 2 and 4b) compared to much larger species (like *Helicosphaera* spp., *S. apsteinii*) which also increased during spring (Figs. 2, 3 and 4b). Another reason could be related to differences in coccolith residence time before reaching the trap at 1200 m, between the two events. Stoll et al.^32^ report on unchangeable and/or attenuated Sr/Ca signals during high coccolith export periods in the eastern Mediterranean as suggestive of increased export efficiency/scavenging. Indeed, Eliason and Seget^34^ have reported that both the upper trap at 1200 (studied here) and the lower trap at 3500 m at mooring site M4 intercepted the export event U24 during the same 16-day interval (data not shown). This indicates a high settling velocity (of at least 140 m/day) resulting in rapid settling and little degradation of organic material (up to 51 mg/m^2^/day of organic matter; Fig. 4f). Dust particles deposited at the surface usually remain in suspension until the occurrence of phytoplankton blooms producing enough organic matter to form larger-sized aggregates (“marine snow”). Both “marine snow” aggregates and faecal pellets produced by zooplankton are likely to incorporate coccoliths and dust particles, which then act as mineral ballast to accelerate their settling velocities^45–48^. Since the efficiency of mineral ballasting is a function of the magnitude of the surface bloom^17,49,50^, our data suggest that surface productivity was particularly high during U24, followed by unusually fast/efficient export, to which rainfall likely contributed. We propose that the observed attenuated Sr/Ca signal in the autumn event partially resulted from a greater mixture of “freshly produced” coccoliths with high Sr/Ca (more abundant in the small fraction), “aged” coccoliths with lower Sr/Ca related to “background” (lower) productivity conditions prior to this event. Our data suggest that the Sr/Ca ratio may be less suitable as a productivity proxy beyond a certain threshold of dust input and/or when dust is rapidly deposited with rain, during which the resulting accelerated ballasting effect is likely to “mask” the freshly produced coccolith “growth” biogeochemical signature. This might be especially true when the coccolith-CaCO_3_ produced during the bloom is dominated by small size species with lower capacity to fractionate Sr compared to larger species.

### Sr/Ca as proxy of productivity in stratified tropical ocean conditions

Some of the highest absolute and normalized Sr/Ca ratios observed during this study clearly coincided with coccolith- and coccolith-CaCO_3_ flux maximum by deep-dwelling taxa, which were by far the most abundant coccolith sinking species during the entire sampling period (Fig. 4c) and contributing to some of the highest coccolith fluxes ever recorded in the open Atlantic Ocean^9^. This was the case under the heavily stratified conditions typical of the ITCZ-influenced summer season when the fluxes of *F. profunda* and *G. flabellatus* co-increased with the Sr/Ca of the bulk, intermediate and large fractions in July 2013 (U18; Figs. 3a and 4a,b,c). The occurrence of a notable increase of dust fluxes during this period (Fig. 4e) suggest that atmospheric nutrients could have contributed to fuel export production in the LPZ. However, recent observations from the photic zone in the tropical NE Atlantic suggest that only opportunistic surface-dwelling species are likely to benefit from pulsed dust-born nutrient-enrichment^10^. For example, while the abundance of *E. huxleyi* and *G. oceanica* was seen increasing in response to the input of iron and phosphorous supplied by Saharan dust deposition off NW Africa (10–15°N), neither the K-selected UPZ species (more typical of the subtropical gyre (consult the Glossary in the Supplementary Material) nor the deep-dwelling communities had any ecological response. In the same way, despite the slight flux increase of *E. huxleyi* and *G. oceanica* in July 2013 (Fig. 4c), one would expect these species to have a more significant response to dust deposition compared to the tropical assemblage, as observed during the spring and autumn dusty events. This was not the case. Instead, high Sr/Ca ratios in summer seemed related to stratified conditions favouring productivity of LPZ species thriving near the deep nutricline. Our data are in good agreement with a previous study reporting high Sr/Ca ratios on the *F. profunda* size fraction being concomitant with the onset of increased fluxes of *F. profunda* in the Northern Bay of Bengal, where the coccolith sinking assemblage was also dominated by an unusually high degree of *F. profunda*^29^. Larger-sized K-selected taxa more typical of the UPZ, which also increased during this period (e.g., *Helicosphaera* spp. and most of the species within the group “other taxa”, several of which of larger size and related greater capacity for Sr incorporation compared to *E. huxleyi* and *G. oceanica*, as discussed below) may have also contributed to increase the Sr/Ca during summer (Fig. 4b,c,d).

### Coccolith size fractions and species-specific Sr/Ca signal

While both field and culture studies show that the Sr/Ca measured in coccolith calcite can be used as a proxy for variations in the rates of growth/calcification rates of coccolithophores (e.g.,^20,24,25,30,31,42,51^), there is a crucial species-specific size control of Sr coccolith content^25^. Our data clearly support this, based on the much higher Sr/Ca ratios measured in the large (> 6 μm) coccolith size fractions. Sr/Ca ranges as high as 1.6–12.6 in this fraction (Table 1) have never been reported in natural samples before and are most likely related to the higher calcite content in larger-sized coccolith species, which are more typical of stratified ocean conditions like those of site M4. In our data, this is mainly driven by the CaCO_3_ contribution of *S. apstenii* and *Pontosphaera* spp. which have extremely high bioaccumulation of Sr compared to other species^41^, but also of *Helicosphaera* spp., and *C. leptoporus* which had their higher percentages in the intermediate and large fractions (Fig. 2).

This is in line with the existing notion that larger-sized and more robust coccoliths (i.e., higher amount of coccolith-CaCO_3_) incorporate more Sr during the cells’ growth, regardless of the degree to which they are ecologically responsive to nutrient-enrichment. Previous studies also describe coccolith Sr partitioning to be variable among genera and species, with larger and more heavily calcified coccoliths usually having higher Sr content compared to smaller and lighter coccoliths^20,25,32,52^. An especially illustrative example of this is the rare but very large species *S. apsteinii*; despite its very low export productivity^17^ and contributing less than 2% of the coccolith assemblages in all size fractions (Fig. 4d and Fig. I—Supplementary Material), this species produced disproportionally high percentages of carbonate both in the original trap samples and in the coccolith size fractions (e.g., samples U18 and U21) (Figs. 2 and 4b). According to coccolith biometric data presented in^17^, coccoliths of *S. apsteinii* were by far the largest coccoliths measured in samples from trap M4 (mean length of 15.24 μm), resulting in a coccolith calcite mass of 1665.06 pg. (Table IV in the Supplementary Material). Previous studies have also reported *S. apsteinii* to have unusually high, and still poorly understood, coccolith Sr/Ca ratios. Hermoso et al.^40^ highlights the unique ability of *S. apsteinii* to strongly fractionate Sr, resulting in much higher Sr/Ca compared to any other coccolithophore species (extant or extinct) for which this ratio has been determined. This results in high Sr/Ca ratios not always coinciding with maxima in export fluxes, in line with observations from previous sediment trap studies in the Sargasso Sea^21,30^ and in the eastern Mediterranean^32^. An example of this is the high Sr/Ca ratio in the small-size fraction co-occurring to low export production in late March 2013 (U10), when some of the lowest total coccolith- and coccolith-CaCO_3_ fluxes of the entire trap time-series were recorded (Fig. 3), including those produced by the dominant *F. profunda* and *G. flabellatus* (Fig. 4b,c). A similar example occurred in late May 2013 (U14), when high Sr/Ca ratios obtained from all size fractions coincided with a period of moderate coccolith export production and low coccolith-CaCO_3_ fluxes (Fig. 3). It could be that the Sr/Ca peaks observed during these periods represent the “tail” of the previous high productivity period (winter event—U5–U9, and spring event—U12, prior to March and May, respectively), as hypothesized in previous studies^30,32^.

The observed correlations between Sr/Ca and several of the most important export production events recorded in late January, mid-April, and late July, suggest an overall efficient coupling between enhanced nutrient-stimulated production in the upper photic zone and coccolith export along the uppermost 1200 m of the ocean. However, given the higher abundance of K-selected large-size coccolith species in typical open-ocean tropical settings such as the studied region, we recommend framing the Sr/Ca data within a multi-proxy approach for more accurately extracting its ecological significance. This should particularly be the case in the presence of *S. apsteinii* and *Pontosphaera* spp. Previous authors have also alerted to the yet several aspects that remain poorly understood about the drivers of Sr incorporation by ecological- and morphologically distinct coccolithophore species (e.g.^41^). For example, we find that the Sr/Ca of all the studied size fractions from trap M4 were generally higher during spring and summer (Figs. 3 and 4a), despite the Sr partitioning having been previously reported as not being affected by changes in growth and calcification rates related to light conditions^25,28,51^. Whether or not seasonal variations in light conditions during the monitored period also played a role in stimulating the biogeochemical incorporation of Sr remains an open question.

## Conclusions

Our synoptic observations of seasonally resolved coccolith Sr/Ca ratios from a sediment trap mooring located in the western tropical North Atlantic during 2012–2013 contribute to understanding the ratio’s potential as a proxy for the biogeochemical effects of Saharan dust deposition in a heavily stratified ocean region. The main conclusions are as follows:

- Strong correlation between Sr/Ca ratios and pulsed increase of coccolith- and coccolith-CaCO_3_ fluxes during the windier, cooler, and high MLD conditions of winter and spring support previous observations of nutrient-stimulated export productivity in the photic zone during these periods.
- The Sr/Ca peak during the pulsed and CaCO_3_-dominated productivity event of mid-April 2013 coincided with the occurrence of dry dust deposition, in line with a scenario of Saharan dust contributing to stimulate the biological carbon pump through providing nutrients to fuel the surface coccolithophore community in the tropical North Atlantic.
- Attenuated Sr/Ca during the pulsed and bSiO_2_-dominated productivity event in October–November 2013 coincided with the occurrence of seasonal inflow of nutrient-enriched Amazon water and wet dust deposition, suggesting that: (a) “freshly produced” coccoliths were, to some degree, diluted with “aged” coccoliths and rapidly dragged/scavenged by dust-induced accelerated sinking velocities; and (b) Sr incorporation was lower due to the small size species *E. huxleyi* and *G. oceanica* being the main coccolith-CaCO_3_ producers during this event.
- High Sr/Ca ratios during maxima of typical tropical taxa, both surface- and deep-dwelling species, during the ITCZ-influenced summer season, validate the ratio as a biogeochemical proxy for enhanced productivity under the heavily stratified ocean conditions typical of tropical open-ocean settings.
- Small-size coccolith fractions, where coccolith-CaCO_3_ was dominated by highly productive but small-sized species *F. profunda*, *G. flabellatus* and *E. huxleyi*, usually presented lower Sr/Ca compared to the other fractions, particularly the large-size fractions which presented unusually high Sr/Ca, to which the abnormally Sr-rich species *Scyphosphaera apsteinii* likely contributed. This clearly supports the existing notion of larger-sized coccoliths being able to incorporate more Sr during their cells’ growth, regardless of the degree to which they are responsive to transient nutrient enrichment.

While the Sr/Ca data confirm the occurrence of previously reported pulsed export productivity in the region, we recommend that the data should be interpreted taking into consideration the carbonate produced by size-distinct species within the coccolith sinking assemblage, and in the context of a multi-proxy framework. Our study suggests that the Sr/Ca ratio is less suitable to be used as a dust-related productivity proxy beyond a certain threshold of dust flux and/or when dust is deposited with rain, during which the dust ballasting effect may contribute to attenuate the coccolith biogeochemical “growth” original signal. Results from this study highlight the importance of doing more research addressing the physiological, biogeochemical, and abiotic drivers of Sr incorporation by ecologically- and morphologically distinct coccolithophore species.

### Supplementary Information

Supplementary Information.

## Data Availability

The original contributions presented in the study are included in the article/Supplementary Material. Further inquiries can be directed to the corresponding author.
