# Publisher Correction: Coccolith-calcite Sr/Ca as a proxy for transient export production related to Saharan dust deposition in the tropical North Atlantic

**DOI:** 10.1038/s41598-024-56736-5

**Published:** 2024-03-14

**Authors:** C. V. Guerreiro, P. Ziveri, C. Cavaleiro, J.-B. W. Stuut

**Affiliations:** 1 MARE - Marine and Environmental Sciences Centre/ARNET - Aquatic Research Network, Faculty of Sciences of the University of Lisbon (FCUL), Lisbon, Portugal; 2 IDL, Instituto Dom Luiz, FCUL, Lisbon, Portugal; 3 Department of Plant Biology, FCUL, Lisbon, Portugal; 4 ICREA, Catalan Institution for Research and Advanced Studies, Barcelona, Spain; 5 ICTA-UAB, Institut de Ciència i Tecnologia Ambientals—Universitat Autònoma de Barcelona, Barcelona, Spain; 6 Marine Geology and Georesources (DivGM), IPMA – Portuguese Institute for Sea and Atmosphere, Lisbon, Portugal; 7 Department of Ocean Systems, NIOZ Royal – Netherlands Institute for Sea Research, Den Burg, The Netherlands; 8 Faculty of Earth and Life Sciences, Vrije Universiteit (VU), Amsterdam, The Netherlands

Correction to: *Scientific Reports* 10.1038/s41598-024-54001-3, published online 21 February 2024

The original version of this Article contained a repeated error, where all instances of the unit ‘µm’ were incorrectly given as ‘mm’.

As a result, in the Material and methodology section, under the subheading ‘Quantification of the coccolith‑Sr/Ca ratios’,

“The laboratory procedure is further described in the Supplementary Material. We present the Sr/Ca ratios determined from the bulk fraction (< 20 mm) and from the three coccolith size-fractions (> 6 mm, 3–6 mm and < 3 mm), which we compared to published data concerning the total and species-specific coccolith- and coccolith-CaCO_3_ export production from the same samples.”

now reads:

“The laboratory procedure is further described in the Supplementary Material. We present the Sr/Ca ratios determined from the bulk fraction (< 20 µm) and from the three coccolith size-fractions (> 6 µm, 3–6 µm and < 3 µm), which we compared to published data concerning the total and species-specific coccolith- and coccolith-CaCO_3_ export production from the same samples.”

Additionally, in the Results, under the subheading, ‘Coccolith CaCO_3_ size fraction separation for Sr/Ca analyses’,

“The bulk fraction (< 20 mm) overall mimicked the seasonal variation of the original coccolith sinking assemblages and related coccolith-CaCO_3_ reported by^8,17^. The small fraction (< 3 mm) was dominated (38–87%) by CaCO_3_ from deep-dwelling species *Gladiolithus flabellatus* and *F. profunda.* The intermediate (~ 3–6 mm) and large fractions (> 6 mm) were dominated by carbonate produced by *Helicosphaera* spp. (up to 83%) followed by *Scyphosphaera apsteinii* (up to 44%), *Calcidiscus leptoporus* (up to 19%) and *Pontosphaera* spp. (up to 7%).”

now reads:

“The bulk fraction (< 20 µm) overall mimicked the seasonal variation of the original coccolith sinking assemblages and related coccolith-CaCO_3_ reported by^8,17^. The small fraction (< 3 µm) was dominated (38–87%) by CaCO_3_ from deep-dwelling species *Gladiolithus flabellatus* and *F. profunda.* The intermediate (~ 3–6 µm) and large fractions (> 6 µm) were dominated by carbonate produced by *Helicosphaera* spp. (up to 83%) followed by *Scyphosphaera apsteinii* (up to 44%), *Calcidiscus leptoporus* (up to 19%) and *Pontosphaera* spp. (up to 7%).”

, And under the subheading ‘Seasonal distribution of the Sr/Ca ratios’.

“We found generally higher ranges of Sr/Ca in the large fraction (> 6 mm; 1.6–12.6), followed by the bulk (< 20 mm; 1.2–5.4) and intermediate-size fractions (3–6 mm; 0.7–5.7), and finally the small size fraction (< 3 mm) with the lowest range (1–2.5).”

now reads:

“We found generally higher ranges of Sr/Ca in the large fraction (> 6 µm; 1.6–12.6), followed by the bulk (< 20 µm; 1.2–5.4) and intermediate-size fractions (3–6 µm; 0.7–5.7), and finally the small size fraction (< 3 µm) with the lowest range (1–2.5).”

Furthermore, in the legend of Fig. 2,

“Species-specific coccolith-CaCO_3_ contribution (%) in the bulk fraction (< 20 μm) and coccolith size fractions (small < 3 mm; intermediate 3–6 mm; and large > 6 mm), from selected sediment trap M4 samples U2, U7, U12, U14, U18, U21 and U24.”

now reads:

“Species-specific coccolith-CaCO_3_ contribution (%) in the bulk fraction (< 20 μm) and coccolith size fractions (small < 3 μm; intermediate 3–6 μm; and large > 6 μm), from selected sediment trap M4 samples U2, U7, U12, U14, U18, U21 and U24.”

In the legend of Fig. 3,

“(**a**) Seasonal variation of the coccolith-Sr/Ca ratios in the bulk fraction (< 20 μm) and in the coccolith size fractions (small < 3 mm; intermediate 3–6 mm; large > 6 mm); (**b**) total coccolith- and coccolith-CaCO_3_ fluxes^8,17^ from sediment trap M4.”

now reads:

“(**a**) Seasonal variation of the coccolith-Sr/Ca ratios in the bulk fraction (< 20 μm) and in the coccolith size fractions (small < 3 μm; intermediate 3–6 μm; large > 6 μm); (**b**) total coccolith- and coccolith-CaCO_3_ fluxes^8,17^ from sediment trap M4.”

And in the legend of Fig. 4,

“(**a**) Normalized coccolith-Sr/Ca ratios from the bulk fraction < 20 mm (light orange line) and coccolith size fractions (small, intermediate and large size fractions—red, blue and black lines, respectively);”

now reads:

“(**a**) Normalized coccolith-Sr/Ca ratios from the bulk fraction < 20 μm (light orange line) and coccolith size fractions (small, intermediate and large size fractions—red, blue and black lines, respectively);”

and,

“Numbers refer to samples U2, U7, U12, U14, U18, U21 and U24, in which we performed a taxonomic analysis of the bulk fraction (< 20 mm) and of the coccolith small, intermediate, and large size fraction (shown in Fig. 2).”

now reads:

“Numbers refer to samples U2, U7, U12, U14, U18, U21 and U24, in which we performed a taxonomic analysis of the bulk fraction (< 20 μm) and of the coccolith small, intermediate, and large size fraction (shown in Fig. 2).”

in the Discussions section, under the subheading ‘Coccolith size fractions and species‑specific Sr/Ca signal’,

“Our data clearly support this, based on the much higher Sr/Ca ratios measured in the large (> 6 mm) coccolith size fractions.”

now reads:

“Our data clearly support this, based on the much higher Sr/Ca ratios measured in the large (> 6 μm) coccolith size fractions.”

and,

“According to coccolith biometric data presented in^17^, coccoliths of *S. apsteinii* were by far the largest coccoliths measured in samples from trap M4 (mean length of 15.24 mm), resulting in a coccolith calcite mass of 1665.06 pg. (Table IV in the Supplementary Material).”

now reads:

“According to coccolith biometric data presented in^17^, coccoliths of *S. apsteinii* were by far the largest coccoliths measured in samples from trap M4 (mean length of 15.24 μm), resulting in a coccolith calcite mass of 1665.06 pg. (Table IV in the Supplementary Material).”

Finally, the header row of Table 1 has been corrected.

**Table:** 

Coccolith Sr/Ca (m mol/mol)			
< 20 micron	< 3 micron	3–6 micron	> 6 micron

now reads:

**Table:** 

Coccolith Sr/Ca (m mol/mol)			
< *20* μm	< *3* μm	*3–6* μm	> *6* μm

The original version of this Article has been corrected.

