# Supplementary material for: Coccolith-calcite Sr/Ca as a proxy for transient export production related to Saharan dust deposition in the tropical North Atlantic
Source: Sci Rep. 2024 Feb 21;14:4295. doi: 10.1038/s41598-024-54001-3 (PMC10881577; doi:10.1038/s41598-024-54001-3)
Supplement: Supplementary file 1 — Supplementary Information. [file 41598_2024_54001_MOESM1_ESM.docx]

**Coccolith-calcite Sr/Ca as a proxy for transient export production**

**related to Saharan dust deposition in the tropical North Atlantic**

Guerreiro, V. C.^1,2,3*^, Ziveri, P.^4,5,^ Cavaleiro, C.^6^, Stuut, J.-B.W.^7,8^

^1^ MARE - Marine and Environmental Sciences Centre/ARNET - Aquatic Research Network, Faculty of Sciences of the University of Lisbon (FCUL), Lisbon, Portugal

^2^ IDL, Instituto Dom Luiz, FCUL, Lisbon, Portugal

^3^ Department of Plant Biology, FCUL, Portugal

^4^ ICREA, Catalan Institution for Research and Advanced Studies, Barcelona, Spain

^5^ ICTA-UAB, Institut de Ciència i Tecnologia Ambientals—Universitat Autònoma de Barcelona, Barcelona, Spain

^6^ IPMA – Portuguese Institute for Sea and Atmosphere, Marine Geology and Georesources (DivGM), Lisbon, Portugal.

^7^ NIOZ Royal – Netherlands Institute for Sea Research, Department of Ocean Systems, Den Burg, The Netherlands

^8^ Faculty of Earth and Life Sciences, Vrije Universiteit (VU), Amsterdam, Netherlands,

*Corresponding author: cataguerreiro@fc.ul.pt

**SUPPLEMENTARY MATERIAL**

**Glossary**

Export production: while coccolithophore production (cells/L) is primarily a function of light and nutrients, modulated by seasonal variations in hydrological parameters like temperature, salinity, turbulence and turbidity, coccolith export production, or coccolith fluxes (coccoliths m^-2^ d^-1^), results from this primary signal, plus the effects from being further affected by several taphonomical phenomena (i.e., necrolysis and biostratinomy) that act upon the coccospheres/coccoliths before they sink out of the upper ocean. Sediment trap studies using this group of calcifying phytoplankton are based on the assumption that the settling of coccoliths in open-ocean areas is primarily related to the production occurring in the overlying photic layer, allowing for the assessment of the export fluxes and seasonal trends of distinct coccolithophore species during longer periods than most plankton studies [1].

Coccolith flux: refers to the number of coccoliths per square meter per day. Coccolith species counts are converted into coccolith export fluxes (i.e. coccoliths m^−2^ d^−1^) by extrapolating to the entire effective filter area and to the original sample, and dividing by the sample interval and the sediment trap aperture area. These fluxes provide a measure of the export production by any given coccolith species.

Coccolith-CaCO_3_ flux: refers to the flux of CaCO_3_ associated to the coccolith, such that coccolith species of different sizes and shapes translate into different fluxes of Coccolith-CaCO_3_. The coccolith-CaCO_3_ flux is calculated based on the coccolith mass equation of [2], according to which the coccolith mass of distinct species is expressed as: **Coccolith calcite (pg) = 2.7 × Ks × l^3^**, in which 2.7 = density of calcite (CaCO_3_); ks = shape constant; l = coccolith size (mostly distal shield length). The obtained coccolith-CaCO_3_ mass for a certain species is then multiplied by its respective coccolith flux in order to obtain its Coccolith-CaCO_3_ flux.

K-selected and r-selected taxa: This ecological terminology was defined by [3] – known as “Margalef Mandala“– stating that the main functional groups of marine phytoplankton are distributed in the ocean in relation to gradients of turbulence and nutrient availability. “K - selected” taxa are typically better adapted to compete successfully for limited nutrient availability in more stable environments, such as tropical regions and subtropical gyres, within populations that are at or near equilibrium conditions for long periods of time. In turn, “r-selected” are more apt at quickly responding to short-term changes associated with nutrient input, and usually more abundant in unstable and nutrient-enriched conditions, such as turbulent coastal environments. Concerning coccolithophores have evolved towards adapting to both types of ocean conditions, depending on the species, which means they include both r- and K-selected taxa within the group.

Dry vs. wet aeolian dust deposition: the importance of addressing whether the surface of the ocean was subjected to dry dust deposition (as in dry fallout of dust particles) or to wet dust deposition (as in wet deposition by rain) has to do with existing evidence that the latter provides a more efficient source of bioavailable (soluble) nutrients for phytoplankton (and thus coccolithophores) uptake compared to “dry deposition”. This happens as a result from dust particles being exposed to cloud processes and mixing with anthropogenic species such as HNO_3_ in the atmosphere [4,5].

**Sediment trap mooring M4 (12ºN/49ºW): environmental background**

Previous studies have already extensively described the oceanographic and meteorological settings, as well as the seasonal patterns of particle fluxes at mooring trap site M4 during the sampling period studied here [1, 6–9]. Water masses influencing the photic zone in this region include the warm, salty, and nutrient-depleted Tropical Surface Water (TSW) in the uppermost ~100 m depth, over the cooler and relatively nutrient-enriched South Atlantic Central Water (SACW) extending down to ∼500 m depth. Surface circulation involving these water masses is mostly driven by the North Equatorial Current (NEC) flowing westwards along ~10-20ºN, forced by the NE trade winds. This, in combination with the Coriolis effect leads to geostrophic deepening of the thermocline from east to west across the tropical North Atlantic basin [10–11], resulting in persistently stratified and deep-nutricline ocean conditions at site M4 [7]. South of the NEC is the North Equatorial Counter Current (NECC) flowing in eastward direction, counter to the wind [13]. Both the NEC and the NECC are subjected to the latitudinal seasonal migration of the Intertropical Convergence Zone (ITCZ) between ~5ºS and 12ºN [14] which, in turn, contributes to seasonally modulate the patterns and composition of the particle fluxes [1,6,7]. In winter and spring, the ITCZ is typically displaced towards the equatorial region resulting in dry and windy conditions over the studied region. In summer and autumn, the ITCZ migrates towards its northernmost position, thereby weakening the trade winds and inducing rainfall over site M4. At the same time, a part of the northward-flowing North Brazilian Current (NBC) retroflects off the NE South American margin towards the east during this time of the year [15], feeding into the NECC [16,17]. This leads the NBC to carry Amazon-driven buoyant waters into the eastward-flowing NECC, resulting in the entrainment of large amounts of nutrients [18–20] onto the surface water masses of the central tropical North Atlantic [21–23]. In parallel, Saharan dust fluxes in the western tropical North Atlantic are also higher in summer [24], mainly transported by the Saharan Air Layer [25–27].

**Coccolith mass separation by the repeated decanting procedure**

The laboratory procedure started with resuspending and disaggregating the sediment-trap particle samples with a buffered solution of pH 10-11 (2 L of distilled water + 30 mL NH_3_), after which each sample solution was split into 10 aliquots using a McLane wet sample rotary divider (deviations between aliquots < 5%). Three of these splits were mixed together and wet-sieved for obtaining three distinct particle-size fractions: 150-63 μm, 63-20 μm and <20 μm filtered on a polycarbonate membrane filter (0.4 μm pore size, 47 mm diameter). From the remaining splits, five were mixed together and wet-sieved for obtaining the < 20 μm bulk fraction, which was then subjected to several cycles of repeated decanting for gravitational separation of large-, intermediate- and small-sized fractions of coccoliths (i.e., large >6 μm, intermediate 3-6 μm, and small <3 μm). All decanting experiments were executed by allowing suspensions to settle in 50-ml centrifuge tubes and pipetting off the desired fractions of supernatant, using Stoke’s Law of settling as an approximation for the relative settling times of different-sized particles (e.g. [18]). Six size-fractions per sediment-trap sample resulted in a total of 138 polycarbonate membrane filters. The filters and particulate material were leached in 10 mL 1% HNO_3_ solution and further placed in the ultrasonic bath for 10 minutes and left to stand for 1 night, after which they were measured for Ca and Sr by ICP-OES spectrometry using a Perkin-Elmer, model Optima 4300DV at ICTA. Different replicate injections were done using the same sample to estimate the injection precision, such that values from 0.1 to 1.3 % of rsd (relative standard deviation) were obtained for Sr and Ca.

**
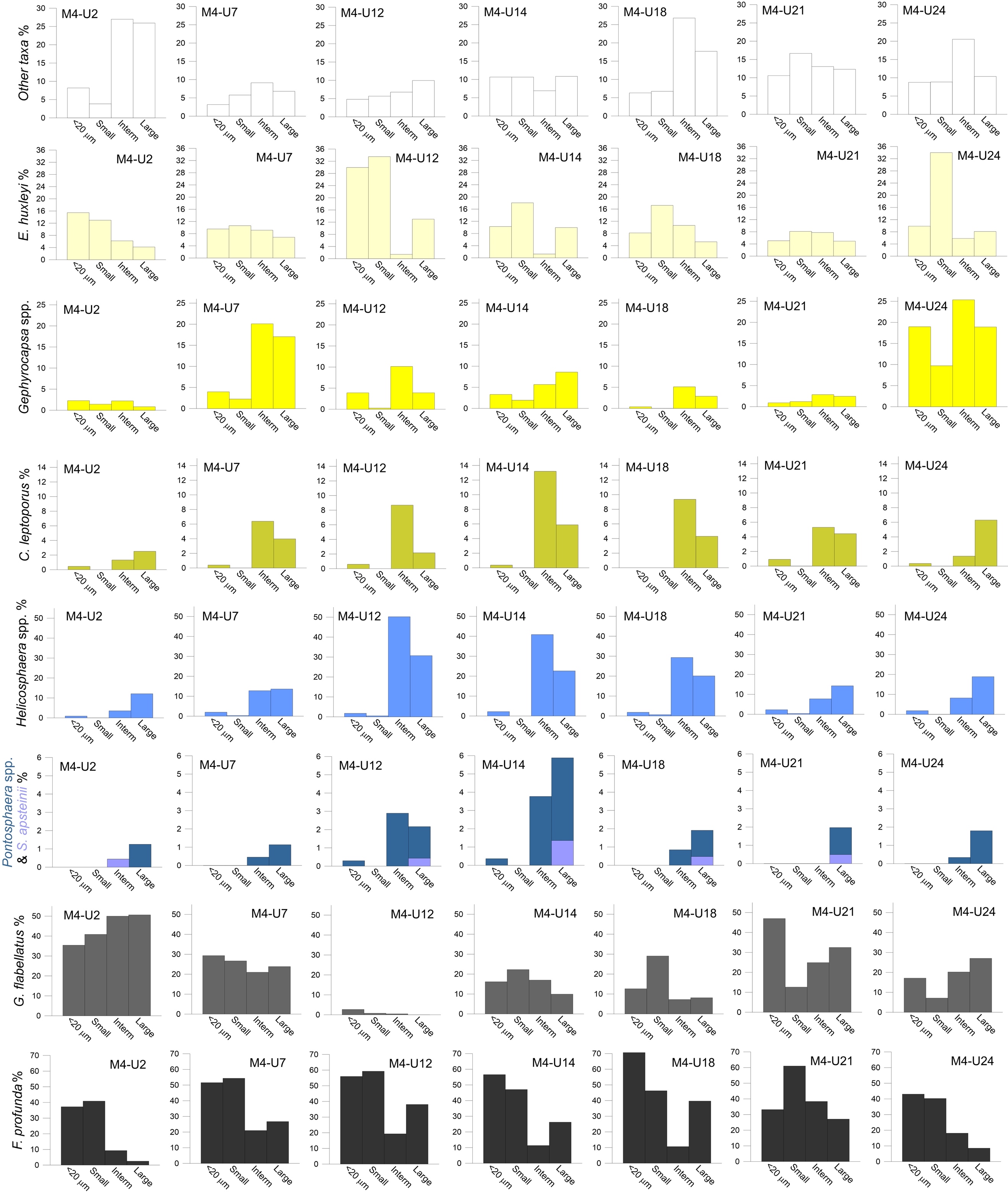
**

**Figure I - Coccolith species percentages in the bulk <20 μm fraction and coccolith suspensions (small <3 mm; intermediate 3-6 mm; and large >6 mm) obtained from sediment trap M4 samples U2, U7, U12, U14, U18, U21 and U24. “Other taxa” include *Umbilicosphaera* spp., *Syracosphaera* spp., *S. pulchra*, *Rhabdosphaera* spp., *Umbellosphaera* spp., *D. tubifera*, *C. mediterranea*, *R. sessilis*, *Calciosolenia* spp. and *C. cristatus* HET. The Sr/Ca were analysed in sediment size fractions dominated by CaCO_3_ produced by coccolithophore species with similar coccolith size and calcite mass. Since Sr incorporation into the coccolith calcite depends on the nutrient-stimulated calcification rate and by the species-specific Sr partitioning (e.g. [28]) the results are interpreted considering these two factors. The <20µm fraction is often used for standard Sr/Ca analyses and offers a relatively easy approach for this type of study. Small-sized coccolith species *E. huxleyi* were generally more frequent in the small size coccolith suspension in all studied samples, but more noticeable in mid-April and October-November 2013 (samples U12 and U24, respectively). *Gephyrocapsa* spp. were, in turn, more frequent in the intermediate- and large-size coccolith suspensions, also more evident during the same periods when coccoliths of *E. huxleyi* were more abundant. Larger-sized coccolith taxa *C. leptoporus* and *Helicosphaera* spp. were much more frequent in the intermediate- and large-size coccolith suspensions, particularly during spring and summer (samples U12, U14 and U18), while nearly absent in the small-size suspensions. *Pontosphaera* spp. and *S. apsteinii* occurred only sporadically (percentages up to 7% and 2%, respectively) but always in the intermediate- and large-size coccolith suspensions. Like *E. huxleyi*, *F. profunda* was more frequent in the small-size coccolith suspension in all the samples, but also increasing in the large-size suspensions from spring and summer (samples U12, U14 and U18). Of all the observed taxa, *G. flabellatus* revealed the less clear coccolith size distribution pattern, with higher percentages of coccoliths in the intermediate- and large-size suspensions during the autumn (samples U2, U21 and U24) and higher percentages in the small-size suspensions during the winter and spring (samples U7, U14 and U18), while nearly absent in all suspensions from mid-April (sample U12). Other taxa, comprising species of different ecological preferences and coccolith sizes, were generally more frequent in the intermediate- and large-size coccolith suspensions, particularly during the autumn and summer (higher percentages in samples U2, U18 and U24). The compositional differences among the microscopically inspected coccolith size fractions were related to seasonal variations in the coccolith export fluxes of ecologically distinct species during the monitored period at trap site M4 (Figure 2 of the manuscript). The obtained coccolith-size redistribution resulted in the deep-dwelling coccolithophores being the main carbonate contributors in most of the microscopically inspected small-sized suspensions, while *Helicosphaera* spp. *S. apsteinii* and *C. leptoporus* (species with high Sr partitioning coefficient; [29,30] contributed to most of the carbonate in the intermediate- and large suspensions. Fast-blooming r-selected placolith-bearing taxa such as *E. huxleyi* and Gephyrocapsid species increased their carbonate percentages in the bulk and small-size suspensions of the periods during which their export productivity increased at trap M4, i.e., during pulsed export events in late January (U7), mid-April (U12) and October-November 2013 (U24) (Figure 2; [1]). The fact that Sr/Ca was more successfully measured from most of the small-size suspensions compared to the other suspensions (Table 1) probably reflects the amount of available sediment, and thereby of coccolith concentrations. Indeed, deep-dwelling species *F. profunda* and *G. flabellatus* were by far the most abundant species at trap M4, followed by *E. huxleyi* [1], thereby more likely to provide enough, as well as more “freshly preserved”, coccoliths for the geochemical analysis.**

| Parameter | Acronym | Units | Sensor | Product | Resolution | Source | Reference |
| --- | --- | --- | --- | --- | --- | --- | --- |
| Chlorophyll | Chl-*a* | mg m^-3^ | MODIS-Aqua | L2-daily | 1 km | [https://oceancolor.gsfc.nasa.gov/cgi/browse.pl?sen=amhttps://oceancolor.gsfc.nasa.gov/cgi/browse.pl?sen=am](https://oceancolor.gsfc.nasa.gov/cgi/browse.pl?sen=am) | OBPG 2014; [31] |
|  |  |  |  | L3-monthly | 4km | <https://oceancolor.gsfc.nasa.gov/cgi/l3> |  |
| Sea Surface Salinity | SSS | none | Aquarius | L3-daily | 1º | <https://podaac.jpl.nasa.gov/dataset/AQUARIUS_L3_SSS_SMI_DAILY_V4> | [32–34] |
| Mixed Layer Depth | MLD | m | - | NASA Ocean Biogeochemical Model (NOBM) VR2017 | - | <https://data.nasa.gov/d/7b98-jn6x?category=dataset&view_name=NASA-Ocean-Biogeochemical-Model-assimilating-satel> | [35] |

**Table I - List of oceanographic and meteorological parameters obtained from satellite imagery, and respective sensors, downloaded products and resolution of the downloaded data.**

**Table II. Principal Components (PCA, correlation mode; PAST-4.11 software), eigenvalues, and percentage of the total variance explained in the data matrix at site M4 (marked loadings are equal or > 0.3). Variables include the log-normalized carbonate fluxes of the main carbonate producing species, the Sr/Ca ratios, and of other particles fluxes (bSiO_2_, OM, dust) and physical–biological proxies (SSS, Chla, MLD) in one single data matrix. Samples which had no data from at least one of the size fractions were excluded from the analysis (U1, U9, U10 and U23). Fluxes of OM and biogenic silica are from [6], Saharan dust fluxes from [9], species-specific coccolith-CaCO_3_ fluxes from [8], Chla and SSS from [1] and MLD data from [7].**

| **Variable** | **PC 1** | | **PC 2** | **PC 3** |
| --- | --- | --- | --- | --- |
| *E. huxleyi* CaCO_3_ (Ehux) | 0,3 | -0,1 | | 0,3 |
| *Gephyrocapsa* spp. CaCO_3_ (Gephy) | 0,3 | -0,1 | | **-0,3** |
| *C. leptoporus* CaCO_3_ (Clept) | 0,1 | -0,1 | | 0,2 |
| *S. apsteinii* CaCO_3_ (Sapst) | 0,2 | 0,0 | | 0,3 |
| *F. profunda* CaCO_3_ (Fprof) | -0,1 | 0,0 | | 0,4 |
| *G. flabellatus* CaCO_3_ (Gflab) | -0,2 | 0,2 | | 0,4 |
| *Helicosphaera* spp. CaCO_3_ (Helico) | 0,2 | -0,1 | | 0,4 |
| *Pontosphaera* spp. CaCO_3_ (Pontos) | 0,1 | 0,2 | | -0,1 |
| Sr/Ca-Bulk | 0,3 | 0,3 | | -0,1 |
| Sr/Ca-Small | 0,4 | 0,0 | | 0,0 |
| Sr/Ca-Intermediate | 0,3 | 0,3 | | 0,1 |
| Sr/Ca-Large | 0,2 | 0,2 | | **-0,4** |
| Biogenic Silica (bSiO_2_) | 0,3 | **-0,3** | | -0,1 |
| Organic Matter (OM) | 0,3 | **-0,3** | | 0,1 |
| Dust | 0,1 | **-0,3** | | 0,3 |
| Chlorophyll-*a* (Chla) | 0,1 | **-0,4** | | -0,2 |
| Sea surface salnity (SSS) | 0,2 | 0,4 | | 0,1 |
| Mixed layer Depth (MLD) | 0,2 | 0,2 | | 0,0 |
| Eigenvalue | 5,1 | 3,4 | | 2,9 |
| % Variance | 28,5 | 19 | | 15,9 |


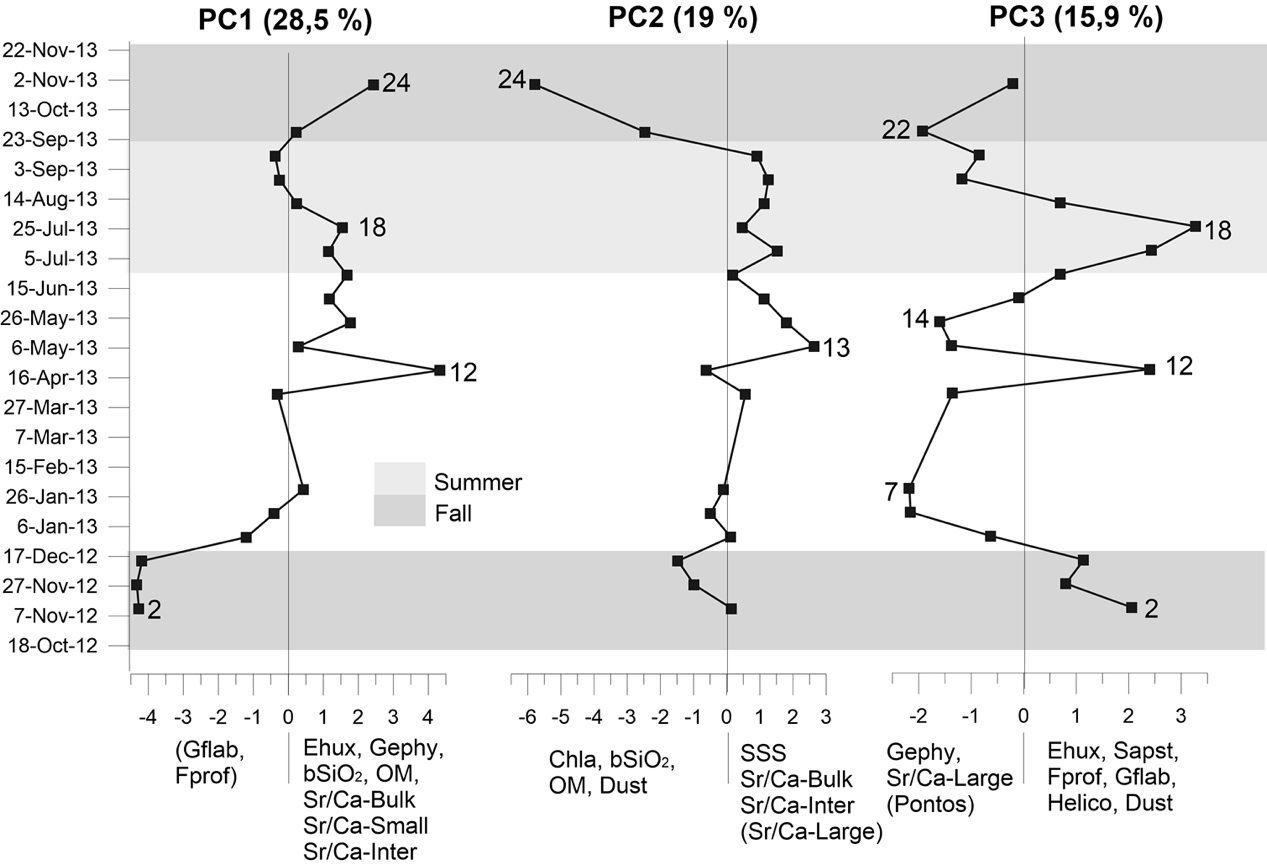


**Figure II. Spatiotemporal variation in the scores obtained from the Principal Component Analysis (PCA, correlation mode; PAST-3 software) performed on a data matrix of 19 samples (cases) and 18 variables (columns). Three principal components (PC1-3) accounted for 63 % of the total variability (Table II). PC1 (28,5%) positive scores represent the correlation between the Sr/Ca of the bulk, small and intermediate fractions, and fluxes of biogenic particles indicative of enhanced productivity in the UPZ at the expense of nutrient-enrichment related to dust deposition during U12 and U24. The latter include fluxes of carbonate produced by r-selected surface-dwelling *E. huxleyi*, and *Gephyrocapsa* spp., and of bSiO_2_ (proxy for diatom export production) and organic matter (OM). Negative scores of PC1 at the start of the studied year coincided with the period during which the Sr/Ca ratios of all fractions were below the annual mean (see Fig. 4a), in line with the observed lower export production by opportunistic surface-dwelling taxa during this period. PC2 (19 %) positive scores appear to be mostly signaling the seasons during which site M4 was not affected by the Amazon River Plume and when Sr/Ca ratios became more persistently above the annual mean (particularly from April to early October 2013). By contrast, PC2 negative scores represent the boreal Autumn periods during which site M4 was being affected by Amazon water, particularly during the very striking event of high surface productivity (Chla) and subsequent export production (bSiO_2_, OM) in October-November 2013 (U24). During this period, the river plume was providing not only nutritious surface water conditions but also buoyancy which allowed to sustain optimum nutrient and light conditions for phytoplankton uptake at the surface. In parallel, Saharan dust was acting both as a source of fertilizing nutrients, which were being retained by the surface river plume, and of mineral ballast which. Combination of high OM production and mineral ballast provided by dust and by opportunistic coccolithophores seems to have induced a particularly efficient export which, in turn, led to a Sr/Ca decrease in all size fractions except the small fraction (see section 5). PC3 (15,9 %) positive scores represent the periods during which the most important coccolith-CaCO_3_ producers were more abundant at site M4, both with and without the influence of dust: deep-dwelling species *F. profunda* and *G. flabellatus* more abundant during the heavily stratified autumn 2012 and summer 2012 (U2 and U18, respectively); *E. huxleyi*, *Helicosphaera* spp. and *S. apsteinii* producing more carbonate during the dry dust deposition event in mid-April 2013 (U12) and, in the case of the latter two taxa, also in July U17-18. Negative scores represent three periods when the Sr/Ca ratio of the large fraction increased to values above the annual mean, two of which coinciding with increased carbonate fluxes by Gephyrocapsid species in January 2013 (U6-7) and, to a lesser extent, by *Pontosphaera* spp. in May-June (U13).**

**Table III. Spearman correlation coefficient matrix showing the relationships between the relative variables included in the PCA data matrix (all the significant correlations are indicated in black and highlighted in different colors according to their strength: light yellow for r = 0.5; orange for r = 0.6; and brown for r ≥ 0.7; default p-level of 0.05). The results generally support the correlations obtained from the PCA, with ecologically similar species displaying strong positive correlations to parameters related to their preferred environmental conditions during the sampling period. For example, deep-dwelling species *G. flabellatus* is positively correlated to F. profunda, but negatively correlated to r-selected *Gephyrocapsa* spp. and to bSiO_2_, though the latter is not statistically significant. Similarly, *E. huxleyi* is positively correlated to *S. apsteinii*, bSiO_2_, OM and SSS, while *Helicosphaera* spp. is positively correlated to dust. Chla shows positive correlations to bSiO_2_, OM and *Gephyrocapsids* and negative correlations to *G. flabellatus*, though not in both cases, the correlations are not statistically significant. Surface-dwelling r-selected taxa such as *E. huxleyi* and *Gephyrocapsa* spp. reveal positive correlations to Sr/Ca ratios of all coccolith size fractions, while deep-dwelling taxa *F. profunda* and *G. fabellatus* are either negatively correlated or not correlated at all with Sr/Ca ratios, even though the majority of these correlations are not statistically significant. Less productive but large-size taxa such as *Heliscosphaera* spp., *S. apsteinii* and *Pontosphaera* spp. also show positive (though not significant) correlations to the Sr/Ca ratio of most of the studied fractions, while *C. leptoporus* showed greater variability, in line with its very low PCA loadings.**

|  | bSiO_2_ | OM | Dust | Chla | SSS | MLD | Ehux | Gephy | Clept | Sapst | Fprof | Gflab | Helico | Pontos | Sr/Ca-Bulk | Sr/Ca-Small | Sr/Ca-Interm | Sr/Ca-Large |
| --- | --- | --- | --- | --- | --- | --- | --- | --- | --- | --- | --- | --- | --- | --- | --- | --- | --- | --- |
| bSiO_2_ |  | 0,8 | 0,2 | 0,4 | 0,3 | 0,5 | 0,5 | 0,7 | 0,4 | 0,2 | 0,0 | -0,4 | 0,1 | 0,0 | 0,2 | 0,6 | 0,1 | 0,2 |
| OM | 0,8 |  | 0,5 | 0,3 | 0,2 | 0,3 | 0,5 | 0,3 | 0,4 | 0,2 | 0,0 | -0,2 | 0,1 | 0,1 | 0,2 | 0,7 | 0,2 | 0,1 |
| Dust | 0,2 | 0,5 |  | 0,1 | -0,2 | -0,3 | 0,3 | -0,1 | 0,4 | 0,2 | 0,0 | -0,1 | 0,6 | -0,2 | 0,1 | 0,5 | 0,3 | -0,3 |
| Chla | 0,4 | 0,3 | 0,1 |  | -0,3 | 0,3 | 0,2 | 0,4 | 0,1 | 0,0 | 0,2 | -0,3 | 0,0 | 0,1 | -0,4 | 0,0 | -0,4 | 0,0 |
| SSS | 0,3 | 0,2 | -0,2 | -0,3 |  | 0,8 | 0,5 | 0,1 | -0,1 | 0,3 | 0,2 | 0,1 | 0,1 | 0,4 | 0,7 | 0,5 | 0,6 | 0,5 |
| MLD | 0,5 | 0,3 | -0,3 | 0,3 | 0,8 |  | 0,4 | 0,2 | 0,1 | 0,2 | 0,4 | 0,1 | 0,0 | 0,5 | 0,3 | 0,2 | 0,1 | 0,3 |
| Ehux | 0,5 | 0,5 | 0,3 | 0,2 | 0,5 | 0,4 |  | 0,3 | 0,1 | 0,5 | 0,3 | 0,2 | 0,4 | 0,0 | 0,4 | 0,4 | 0,3 | 0,1 |
| Gephy | 0,7 | 0,3 | -0,1 | 0,4 | 0,1 | 0,2 | 0,3 |  | -0,1 | -0,1 | -0,1 | -0,5 | 0,0 | -0,1 | 0,0 | 0,2 | 0,0 | 0,4 |
| Clept | 0,4 | 0,4 | 0,4 | 0,1 | -0,1 | 0,1 | 0,1 | -0,1 |  | 0,1 | -0,1 | 0,1 | -0,1 | -0,2 | 0,0 | 0,3 | -0,1 | -0,4 |
| Sapst | 0,2 | 0,2 | 0,2 | 0,0 | 0,3 | 0,2 | 0,5 | -0,1 | 0,1 |  | 0,1 | 0,3 | 0,3 | -0,1 | 0,4 | 0,2 | 0,4 | -0,2 |
| Fprof | 0,0 | 0,0 | 0,0 | 0,2 | 0,2 | 0,4 | 0,3 | -0,1 | -0,1 | 0,1 |  | 0,6 | 0,4 | 0,3 | -0,1 | -0,3 | -0,2 | -0,3 |
| Gflab | -0,4 | -0,2 | -0,1 | -0,3 | 0,1 | 0,1 | 0,2 | -0,5 | 0,1 | 0,3 | 0,6 |  | 0,2 | 0,1 | 0,0 | -0,3 | 0,0 | -0,5 |
| Helico | 0,1 | 0,1 | 0,6 | 0,0 | 0,1 | 0,0 | 0,4 | 0,0 | -0,1 | 0,3 | 0,4 | 0,2 |  | -0,2 | 0,1 | 0,3 | 0,2 | -0,2 |
| Pontos | 0,0 | 0,1 | -0,2 | 0,1 | 0,4 | 0,5 | 0,0 | -0,1 | -0,2 | -0,1 | 0,3 | 0,1 | -0,2 |  | 0,1 | 0,0 | 0,1 | 0,3 |
| Sr/Ca-Bulk | 0,2 | 0,2 | 0,1 | -0,4 | 0,7 | 0,3 | 0,4 | 0,0 | 0,0 | 0,4 | -0,1 | 0,0 | 0,1 | 0,1 |  | 0,6 | 0,9 | 0,5 |
| Sr/Ca-Small | 0,6 | 0,7 | 0,5 | 0,0 | 0,5 | 0,2 | 0,4 | 0,2 | 0,3 | 0,2 | -0,3 | -0,3 | 0,3 | 0,0 | 0,6 |  | 0,7 | 0,3 |
| Sr/Ca-Interm | 0,1 | 0,2 | 0,3 | -0,4 | 0,6 | 0,1 | 0,3 | 0,0 | -0,1 | 0,4 | -0,2 | 0,0 | 0,2 | 0,1 | 0,9 | 0,7 |  | 0,4 |
| Sr/Ca-Large | 0,2 | 0,1 | -0,3 | 0,0 | 0,5 | 0,3 | 0,1 | 0,4 | -0,4 | -0,2 | -0,3 | -0,5 | -0,2 | 0,3 | 0,5 | 0,3 | 0,4 |  |

**Table IV. Biometric data and mass calcite of the coccolithophore taxa found in the bulk fraction and coccolith suspensions (data from samples U2, U12 and U24; [8].**

| **Coccolithophore taxa** | **Coccolith size (μm)** | | **Mass CaCO_3_ (pg)** |
| --- | --- | --- | --- |
|  | Min. - Max. | Mean |  |
| *Emiliania huxleyi* | 2.33 - 3.92 | 3.15 | 2.55 |
| *Gephyrocapsa* spp. |  |  |  |
| *G. oceanica* | 3.09 - 5.56 | 4.22 | 10.16 |
| *G. muellerae* | 2.94 - 4.21 | 3.29 | 4.34 |
| *G. ericsonii* | 2.01 - 2.82 | 2.54 | 2.0 |
| *Calcidiscus leptoporus* | 5.01 - 7.26 | 6.17 | 50.67 |
| *Helicosphaera carteri/wallischiii* | 6.46 - 11.85 | 8.57 | 84.96 |
| *Scyphosphaera apsteinii* | 14.05 - 15.23 | 15.24 | 1665.06 |
| *Florisphaera profunda* | 1.3 - 5.59 | 2.72 | 2.17 |
| *Gladiolithus flabellatus* | 3.74 - 10.97 | 7.07 | 9.65 |
| Other taxa |  |  |  |
| *Calciosolenia brasiliensis* | 4.5 - 6.17 | 5.16 | 12.97 |
| *Ceratolithus cristatus HET* | 6.4 - 10.53 | 8.41 | 47.29 |
| *Coronosphaera mediterranea* | 3.12 - 4.32 | 3.57 | 8.58 |
| *Discosphaera tubifera* | 2.45 - 4.5 | 3.50 | 8.13 |
| *Pontosphaera* spp. | 6.9 - 10.39 | 8.41 | 77.97 |
| *Reticulofenestra sessilis* | 2.75 - 3.55 | 3.18 | 4.33 |
| *Rhabdospahera stylifera* | 4.12 - 6.24 | 5.17 | 14.88 |
| *Syracosphaera pulchra* | 3.56 - 6.75 | 5.16 | 11.1 |
| *Umbellosphaera tenuis* | 3.05 - 7.77 | 5.37 | 8.37 |
| *Umbilicosphaera sibogae* | 3.23 - 4.79 | 4.09 | 11.12 |

**References**

[1] Guerreiro, C.V., Baumann, K.-H., Brummer, G.-J.A., Fischer, G., Korte, L.F., Merkel, U.S.C., de Stigter, H., Stuut, J.-B.W. Coccolithophore fluxes in the open tropical North Atlantic: influence of thermocline depth, Amazon water, and Saharan dust. *Biogeosciences* **14**, 4577–4599, <https://doi.org/10.5194/bg-14-4577-2017> (2017).

[2] Young, J. R., Ziveri. P. Calculation of coccolith volume and its use in calibration of carbonate flux estimates. *Deep-Sea Res. Pt. II* **47**, 1679–1700 (2000).

[3] Margalef, R. Life-forms of phytoplankton as survival alternatives in an unstable environment. *Oceanol. Acta* **1**, 493–509 (1978).

[4] Ridame, C., Dekaezemacker, J., Guieu, C., Bonnet, S., L’Helguen, S., Malien, F. Contrasted Saharan dust events in LNLC environments: impact on nutrient dynamics and primary production. *Biogeosciences* **11** (17), 4783–4800 (2014)

[5] Baker, A.R., Kanakidou, M., Nenes, A., Myriokefalitakis, S., Croot, P.L., Duce, R.A., Gao, Y., Guieu, C., Ito, A., Jickells, T.D., Mahowald, N.M., Middag, R., Perron, M.M.G., Sarin, M.M., Shelley, R., Turner, D.R.. Changing atmospheric acidity as a modulator of nutrient deposition and ocean biogeochemistry. *Sci. Adv.* **7**, eabd8800 (2021)

[6] Korte, L., Brummer, G.-J., van der Does, M., Guerreiro, C., Hennekam, R., van Hateren, J. A., Jong, D., Munday, C. I., Schouten, S. & Stuut, J-B. Downward particle fluxes of biogenic matter and Saharan dust across the equatorial North Atlantic. *Atmos. Chem. Phys.* **17**, 6023–6040, <https://doi.org/10.5194/acp-17-6023-2017> (2017)

[7] Guerreiro, C. V., Baumann, K.-H., Brummer, G.-J. A., Fischer, G., Korte, L.F., Stuut, J.-B.W. Wind-forced transatlantic gradients in coccolithophore species fluxes. *Prog. Oceanogr.* **176**, 102140. <https://doi.org/10.1016/j.pocean.2019.102140> (2019).

[8] Guerreiro, C.V., Baumann, K.-H., Brummer, G.-J. A., Valente, A., Fischer, G., Ziveri, P., Brotas, V., Stuut J.-B. W. Carbonate fluxes by coccolithophore species between NW Africa and the Caribbean: implications for the biological carbon pump. *Limnol. Oceanogr.* **9999**, 1–19, <http://doi.org/10.1002/lno.11872> (2021).

[9] van der Does, M., Brummer, G.‐J. A., van Crimpen, F. C. J., Korte, L. F., Mahowald, N. M., Merkel, U., Stuut, J-B. Tropical rains controlling deposition of Saharan dust across the North Atlantic Ocean. *Geophysical Research Letters* **47** (5), e2019GL086867. <https://doi.org/10.1029/2019GL086867> (2020).

[10] Merle, J. Seasonal heat budget in the equatorial Atlantic Ocean. *J. Phys. Oceanogr.* **10**, 464–469 (1980a).

[11] Merle, J. Seasonal variation of heat storage in the tropical Atlantic Ocean. *Oceanol. Acta* **3**, 455463 (1980b).

[12] Katz, E.J. Dynamic topography of the sea surface in the equatorial Atlantic. *J. Mar. Res.* **39**, 53–63 (1981).

[13] Stramma, L. and Schott, F. The mean flow field of the tropical Atlantic Ocean. *Deep-Sea Res. II* **46**, 279–303 (1999).

[14] Basha, G., P. Kishore, M. Venkat Ratnamc, T. B. M. J. Ouarda, I. Velicogna, T. Sutterley. Vertical and latitudinal variation of the intertropical convergence zone derived using GPS radio occultation measurements. *Remote Sens. Environ.* **163**, 262–269 (2015).

[15] Philander, S.G., Atlantic Ocean Equatorial Currents. *Encyclop. Ocean Sci.*, 188–191. <https://doi.org/10.1006/rwos.2001.0361> (2001).

[16] Richardson, P.L., Walsh, D. Mapping climatological seasonal variations of surface currents in the tropical Atlantic using ship drifts. *J. Geophys. Res*. **91**, 10537–10550 (1986).

[17] Schott, F. A., Fischer, J. and Stramma, L. Transports and pathways of the upper- layer circulation in the western tropical Atlantic. *J. Phys. Oceanogr*. **28**, 1904–1928 (1998).

[18] Boyle, E.A., Edmond, J.M., Sholkovitz, E.R. On the mechanism of iron removal in estuaries. *Geoch.* *Geoph. Acta* **41**, 1313–1324 (1977).

[19] Sholkovitz, E. R., Boyle, E. A., Price, N. B. The removal of dissolved humic acids and iron during estuarine mixing. *Earth Planet. Sc. Lett.* **40**, 130–136 (1978).

[20] De Master, D.J., Kuehl, S.A., Nittrouer, C.A. Effects of suspended sediments on geochemical processes near the mouth of the Amazon River: examination of biogenic silica uptake and the fate of particle-reactive elements. *Cont. Shelf Res.* **6**, 107–125 (1986).

[21] Muller-Karger, F., McClain, C., and Richardson, P. The dispersal of the Amazon’s water. *Nature* **333**, 56–58 (1988).

[22] Molleri, G.S.F., Novo, E.M.L., Kampel, M. Space–time variability of the Amazon River plume based on satellite ocean color. *Cont. Shelf Res.* 30, 342–352 (2010).

[23] Ffield, A. North Brazil current rings viewed by TRMM Microwave Imager SST and the influence of the Amazon Plume, *Deep-Sea Res. Pt. I* **52**, 137–160 (2005).

[24] Prospero, J., Collard, F-X., Molinié, J., Jeannot, A. Characterizing the annual cycle of African dust transport to the Caribbean Basin and South America and its impact on the environment and air quality. *Global Biogeochem. Cy*. **29**, 757–773 (2014).

[25] Stuut, J.-B. W. et al. Cruise report and preliminary results (64PE378), TRAFFIC II: Transatlantic fluxes of Saharan dust (Las Palmas de Gran Canaria, Spain – St. Maarten). Royal NIOZ, pp 54 (2013).

[26] Adams, A. M., Prospero, J. M., Zhang, C. CALIPSO‐derived three‐dimensional structure of aerosol over the Atlantic basin and adjacent continents. *Journal of Climate* **25** (19), 6862–6879. <https://doi.org/10.1175/JCLI‐D‐11‐00672.1> (2012).

[27] Tsamalis, C., Chedin, A., Pelon, J., Capelle, V. The seasonal vertical distribution of the Saharan Air Layer and its modulation by the wind. *Atmospheric Chemistry and Physics* **13** (22), 11,235–11,257. <https://doi.org/10.5194/acp‐13‐11235‐2013> (2013).

[28] Stoll, H.M., Ziveri, P. Separation of monospecific and restricted coccolith assemblages from sediments using differential settling velocity. *Marine Micropaleontology* **46**, 209-221 (2002).

[29] Stoll, H.M., Klaas, C.M., Probert, I., Encinar, J.R., Alonso, I.G. Calcification rate and temperature effects on Sr partitioning in coccoliths of multiple species of coccolithophorids in culture. *Global and Planetary Change* **34**, 153–171 (2002b).

[30] Meyer, E.M., Langer, G., Brownlee, C., Wheeler, G.L., Taylor, A.R. Sr in coccoliths of Scyphosphaera apsteinii: Partitioning behavior and role in coccolith morphogenesis. *Geochimica et Cosmochimica Acta* **285**, 41–54. <https://doi.org/10.1016/j.gca.2020.06.023> (2020)

[31] Hu, C., Lee, Z., and Franz, B. A. Chlorophyll-*a* algorithms for oligotrophic oceans: a novel approach based on three-band reflectance difference. *J. Geophys. Res.* **117**, C01011TS34, <https://doi.org/10.1029/2011JC007395> (2012)

[32] NASA Aquarius project. Aquarius Official Release Level 3 Sea Surface Salinity Standard Mapped Image Daily Data V4.0. Ver. 4.0. PO.DAAC, CA, USA (2015a).

[33] NASA Aquarius project. Aquarius Official Release Level 3 Wind Speed Standard Mapped Image Daily Data V4.0. Ver. 4.0. PO.DAAC, CA, USA (2015b).

[34] Lee, T., Lagerloef, G., Gierach, M.M., Kao, H.-Y., Yueh, S., Dohan, K. Aquarius reveals salinity structure of tropical instability waves. *Geophys. Res. Lett*. **39**, L12610 (2012).

[35] Gregg, W., Rousseaux, C., 2017. NASA Ocean Biogeochemical Model assimilating satellite chlorophyll data global daily VR2017, Edited by Watson Gregg and Cecile Rousseaux, Greenbelt, MD, USA, Goddard Earth Sciences Data and Information Services Center (GES DISC), Accessed: June 2019, 10.5067/PT6TXZKSHBW9.
